# Drivers of Ecosystem Metabolism in an Anthropogenically Modified Delta

**DOI:** 10.1007/s12237-026-01833-z

**Published:** 2026-10-23

**Authors:** Martin P. Volaric, Brian A. Bergamaschi, Ariella Chelsky, David B. Senn, Stephen G. Monismith

**Affiliations:** 1 Environmental Fluid Mechanics Laboratory, Department of Civil and Environmental Engineering, Stanford University, Stanford, CA 94305 USA; 2 California Water Science Center, U.S. Geological Survey, Sacramento, CA USA; 3 San Francisco Estuary Institute, Richmond, CA 94804 USA

**Keywords:** Net ecosystem metabolism, Productivity, Primary production, Respiration, Sacramento-San Joaquin Delta, Oxygen

## Abstract

Estuaries are under threat globally from a wide range of stressors, with rates of ecosystem metabolism – respiration (R), gross primary production (GPP), and net ecosystem metabolism (NEM) – representing a way for resource managers to quantify their impacts. In this study, we assessed how natural and anthropogenic stressors have altered metabolism within the extensively modified Sacramento-San Joaquin Delta (“Delta”) in California, USA. Metabolism was estimated using a diel open-water technique utilizing dissolved oxygen measurements from a high frequency (15 min) water quality monitoring array, and spanned 6 sites across 11 years (2014–2025). Our analyses were driven by a conceptual model of Delta carbon (C) cycling focused on 1) Invasive aquatic vegetation (IAV), 2) Phytoplankton, 3) Wastewater treatment plant (WWTP) upgrades, 4) Large-scale flow alterations, and 5) Wet vs. dry years. We found that allochthonous C fuels net heterotrophy in the outer riverine sections of the Delta, while the heavily vegetated central Delta produces autochthonous C remineralized elsewhere in the system. IAV were the dominant primary producers, with low phytoplankton production except during a historic bloom. WWTP upgrades imparted negligible changes to metabolism, with low chlorophyll *a* (< 5 µg L^−1^) both before and after. Introduction of a flow barrier – a management response to drought – hydrodynamically isolated deep-water channels from nearby shallow areas, resulting in decreased metabolism indicative of changes to C transport. Contrary to past research, low rainfall years were associated with increased metabolism. This study demonstrates how multi-sensor arrays can be used to quantify ecosystem responses to major environmental stressors.

**Supplementary Information:**

The online version contains supplementary material available at https://doi.org/10.1007/s12237-026-01833-z.

## Introduction

Estuaries worldwide are impacted by anthropogenic factors including nutrient loading (Pinckney et al., 2001), invasive species (Williams & Grosholz, 2008), altered landscapes (Cloern et al., 2021), and changing weather patterns (Statham, 2012). With this wide range of ecological stressors, it is important for resource managers to develop metrics that can integrate across the entire estuarine environment to assess health and function. Rates of ecosystem metabolism – gross primary production (GPP), respiration (R), and net ecosystem metabolism (NEM) – describe the balance of carbon (C) in an ecosystem and are one such tool, providing insight into ecosystems beyond what can be ascertained from static indicators such as phytoplankton or nutrient levels alone (Young et al., 2008). Estimates of ecosystem metabolism can be used to quantify the response of ecosystems to natural and anthropogenic perturbations (Staehr et al., 2012), including nutrient enrichment or environmental restoration (Jankowski et al., 2021).

In aquatic systems, metabolic rates are often estimated using dissolved oxygen (DO) as a proxy for C fixation and remineralization (Glud, 2008), allowing them to be straightforwardly applied to many existing estuarine datasets. Recent years have seen the proliferation of continuous high-frequency monitoring arrays in aquatic systems, to collect real-time water quality data including DO(Bailey et al., 2019), allowing for the calculation of metabolic rates at multiple locations and across a range of timescales. This high degree of spatiotemporal resolution is important for estimates in dynamic coastal systems such as estuaries and river deltas, which are likely to show substantial metabolic differences in time and space (Gattuso et al., 1998). Among the most widely used methodologies for converting DO measurements to metabolism estimates is the diel open-water method. First developed by Odum (1956) for stream metabolism, this technique estimates GPP, R, and NEM using hourly or sub-hourly temporal changes in DO across a 24 h day (Staehr et al., 2010), and has been shown to be effective in estuarine environments (Caffrey, 2003, 2004).

Measurements of ecosystem metabolism are particularly applicable to heavily modified, highly monitored waterways such as the Sacramento-San Joaquin Delta (hereafter “Delta”) in California, USA. The Delta is a tidally dominated network of channels representing the transition between the Sacramento and San Joaquin Rivers and San Francisco Bay (SFB), within the greater San Francisco estuary (SFE), and is a key source of drinking water to central and southern California, and of freshwater for agriculture within California’s Central Valley (Lund, 2016). Given its position between heavily urbanized and agriculturally-dominated watersheds, the Delta ecosystem is subject to numerous anthropogenic stressors. Landscape change has greatly altered the Delta from its historical state, resulting in a > 90% decrease in net primary production since the early 1800 s (Cloern et al., 2021) coinciding with a shift in the source of organic carbon (OC) from marshes to aquatic plants and phytoplankton (Vaughn et al., 2020). Of particular concern is the expansion of invasive aquatic vegetation (IAV) including Brazilian waterweed (*Egeria densa*), water hyacinth (*Pontederia crassipes*), and water primrose (*Ludwigia* spp), all of which are now ubiquitous throughout much of the region (Cooke et al., 2018; Khanna et al., 2018). These invaders have imposed numerous deleterious impacts on the Delta, such as clogging waterways, trapping sediment, and providing habitat for invasive predatory fish species (Boyer & Sutula, 2015; Ta et al., 2017). SFE also experiences substantial nutrient loading at levels associated with highly eutrophic systems, which is attributed primarily to wastewater treatment plant (WWTP) discharge and agricultural runoff (Cloern et al., 2020). Although nutrient concentrations are sufficient to support the expansion of IAV in the Delta (Cooke et al., 2018), the precise link between Delta nutrients and macrophytes is poorly understood (Christman et al., 2023). The impacts of nutrients on phytoplankton in the Delta have been somewhat muted as well. Substantial turbidity, flushing rates, and benthic grazing have resulted in generally low phytoplankton productivity despite high nutrient loads, with adverse impacts on food webs that support endangered and threatened endemic fish populations (Cloern, 2001; Jassby et al., 2002; Kimmerer & Thompson, 2014; Moyle et al., 2016).

Flows within the Delta are heavily managed by Federal, State, and local agencies. These flow alterations exist against the backdrop of a California climate defined by large interannual variations in precipitation and river discharge amid an overall pattern of increasing drought severity (Mann & Gleick, 2015). Low freshwater discharge during lower rainfall water years contributes to saltwater intrusion from SFB into the western margins of the Delta, impacting drinking water supply (Durand et al., 2020) and possibly increasing the likelihood of harmful algal bloom (HAB) formation (Bouma-Gregson et al., 2024). To protect drinking water supplies and the Delta ecosystem during drier years, resource managers typically export less water out of the Delta to irrigation districts in the Central Valley and to drinking water agencies south of the Delta (Lund, 2016). An additional tool for the management of Delta salinity is the Delta Cross Channel (DCC) gate, which redirects water from the Sacramento River into the North and South Mokelumne Rivers via a canal. The result is the injection of additional flow into the central Delta to combat tidal influx of salinity. During especially low-flow years or prolonged droughts, emergency drought barriers (EDBs) may also be deployed, as happened in 2015 and 2021 (Hartman et al., 2022). EDBs redirect tidal flows away from drinking water transport corridors through the Delta, substantially altering local hydrodynamics and ecosystem connectivity.

Previous studies of Delta metabolism have either utilized parametric relationships based on chlorophyll (chl) *a* concentration (Jassby et al., 2002), calculated the mass balance of organic matter inputs and outputs (Jassby & Cloern, 2000; Sobczak et al., 2005), or scaled metabolic estimates based on habitat type (Cloern et al., 2021; Cohen et al., 2014; Robinson et al., 2016). These past studies relied on targeted field measurements, intensive modeling of historical data, or in the case of chl *a* analyses, monthly discrete sample collection. Accordingly, unlike DO-based metabolic estimates from high-frequency, continuous data, they are unable to capture transient events, nor are they easily applicable for continuous environmental monitoring. To our knowledge, the only previous diel open-water estimates of Delta metabolism based on water column DO have consisted of relatively short duration (2–3 months) studies within the Sacramento River Deep Water Ship Channel in the northern Delta (Loken et al., 2021; Smits et al., 2024).

In this study, we used continuous monitoring data from a U. S. Geological Survey (USGS) array of high frequency (15 min) water quality sensors to estimate ecosystem metabolism at six sites within the Delta. Metabolic estimates span a more than 10-year period (2014–2025), including historically wet and dry years, allowing us to quantify the impact of physical drivers over monthly and interannual timescales. Our analyses were guided by a conceptual model of OC for the Delta, including predicted impacts from five ecosystem stressors: 1) IAV, 2) Phytoplankton, 3) WWTP upgrades, 4) Large-scale flow alterations including EDBs and DCC flow, and 5) Wet vs. dry years. Our goals were to assess general spatiotemporal patterns and impacts of these stressors on Delta metabolism, while also evaluating the applicability and limitations of DO-based diel metabolic estimates for future monitoring efforts.

## Data and Methods

### Sites and Data Collection

**Fig. 1:**
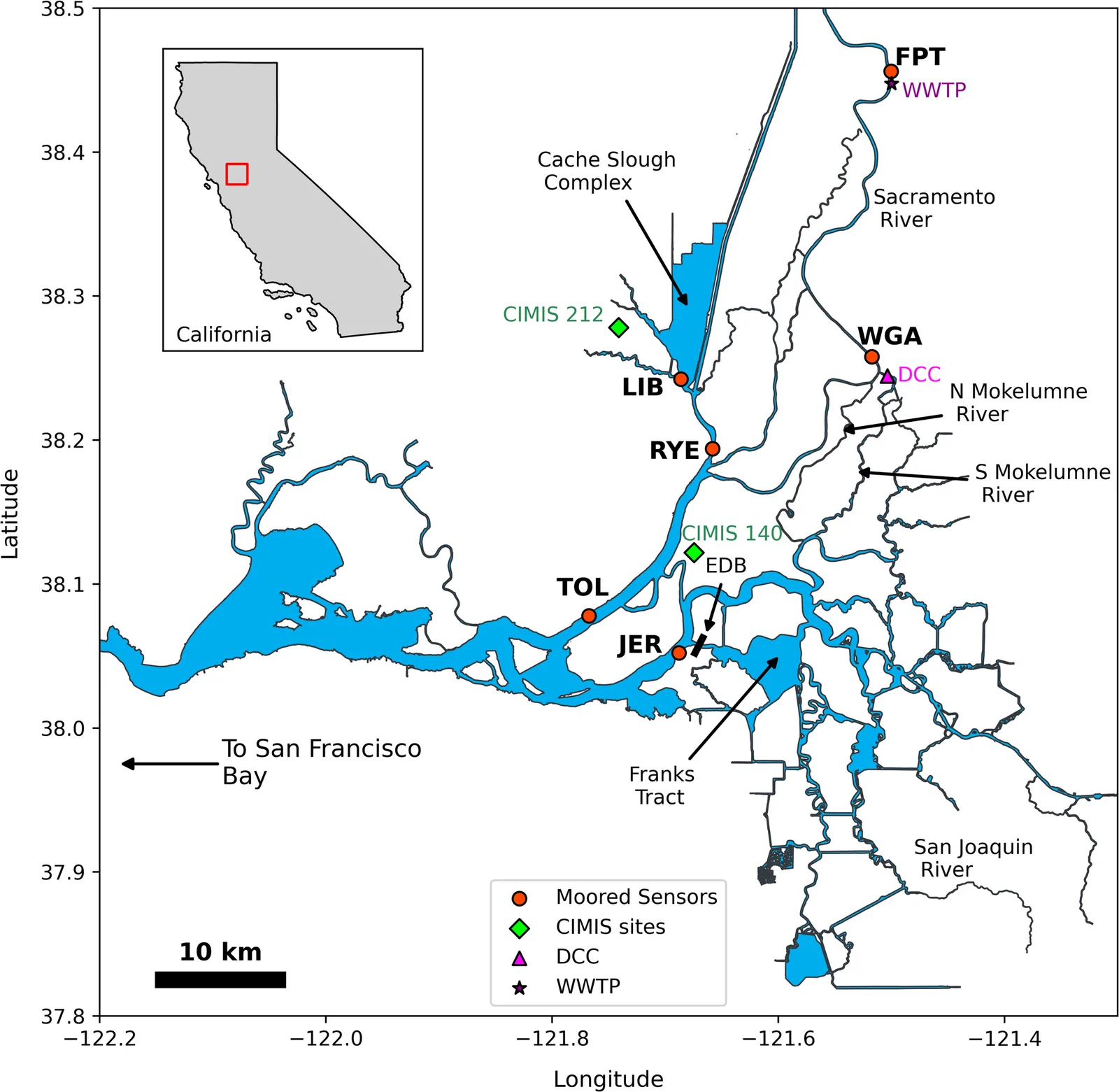
Location of measurement sites in the Sacramento-San Joaquin Delta, which feeds into San Francisco Bay. Six sites were chosen: 2 along the Sacramento River that were predominantly freshwater and had little flow reversal (FPT, WGA), 2 along a mesohaline tidal channel with regular flow reversal (LIB, RYE), and two at mesohaline junctions of several tidal channels (JER, TOL). Also shown are the locations of the heavily vegetated Cache Slough Complex and Franks Tract areas, EchoWater Facility WWTP , Delta Cross Channel (DCC), California Irrigation Management Information System (CIMIS) meteorological sites, and an emergency drought barrier (EDB) that was in place June 2021 – October 2022. Base maps are modified from U.S. Geological Survey and other Federal digital data

Water quality data for this study came from a high-frequency (15 min) USGS moored sensor array deployed within the Delta (Kraus et al., 2017a). The six sites that were chosen for analysis and their respective USGS site IDs included (Fig. 1): 2 riverine locations with minimal tidal impacts – Freeport (FPT—11447650), Walnut Grove (WGA—11447890) – and 4 tidally dominated sites further down the Delta that experience regular flow reversal – Liberty Island (LIB—11455315), Ryer Island (RYE—11455385 & 11455,350), Toland Landing (TOL—11455485 & 11455478), Jersey Point (JER—11337190). LIB and JER are located at the outlets of the Cache Slough complex and Franks Tract, respectively, which are two of the most heavily vegetated regions of the Delta. RYE and TOL are within deep-water (> 8 m) channels with minimal vegetation. These data are publicly available in the USGS National Water Information System (U.S. Geological Survey, 2025), and include DO, chl *a*, water temperature (T), and turbidity (Tu), which were measured using EXO2 multiparameter sondes (YSI Inc./Xylem Inc., Yellow Springs, Ohio), and discharge (Q), which was estimated using site-specific stage-discharge relationships. For our study we also estimated mean water column depth (H) from site-specific stage-area relationships along with channel width: *H=area/width*.

Data was collected during the period Jan 2014 – Mar 2025 for all sites except Jersey Point, which covers the period Sep 2016 – Mar 2025. Hourly windspeed and solar radiation data were taken from the California Irrigation Management Information System (CIMIS) website (California Irrigation Management Information System, 2025)). The nearest CIMIS site was identified and paired with data for each USGS station. CIMIS site #140 (Twitchell Island) was paired with JER and TOL, while CIMIS site #212 (Hastings Tract East) was paired with FPT, WGA, LIB and RYE (Fig. 1).

### Conceptual Model of Delta Metabolism

**Fig. 2:**
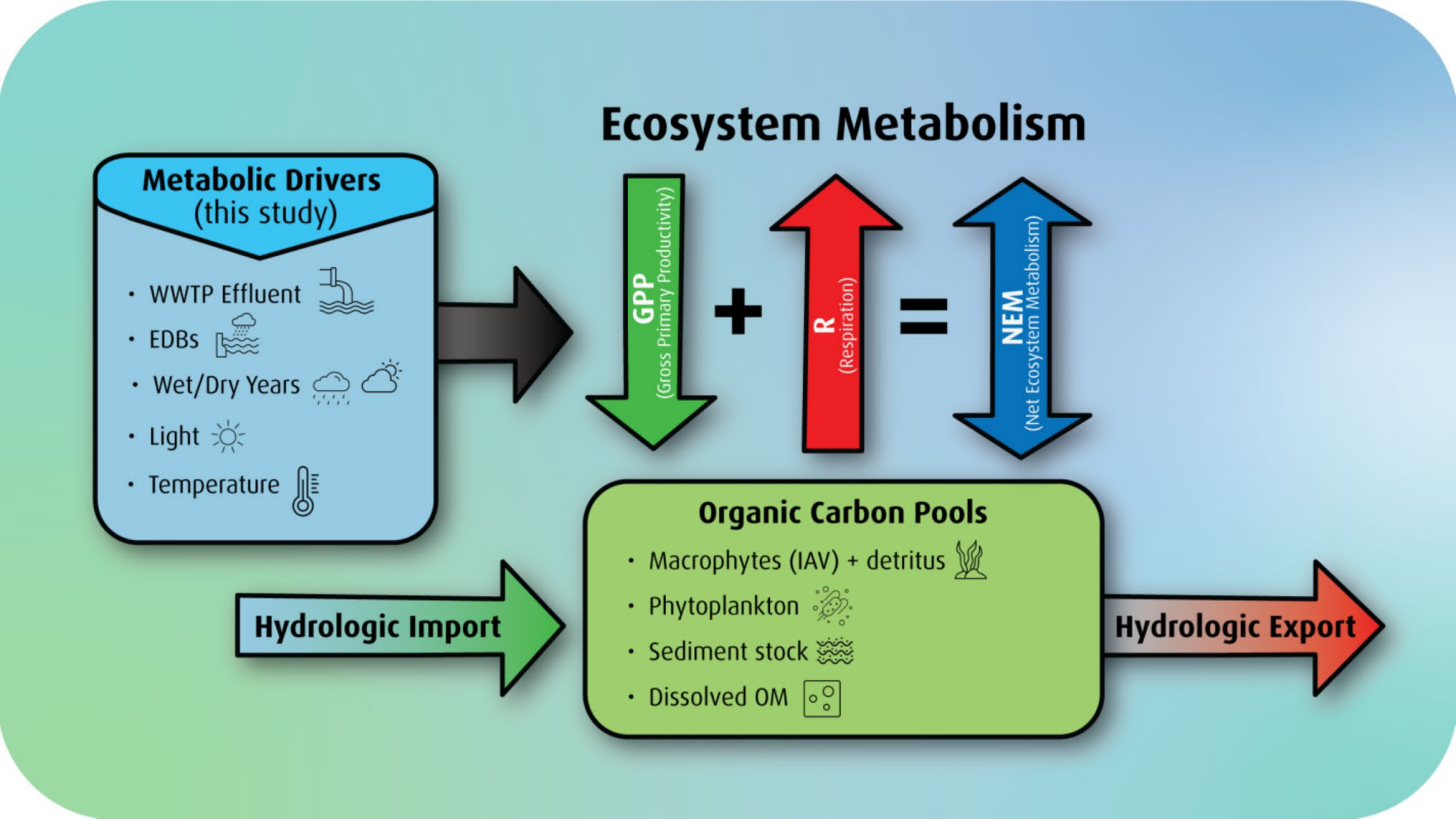
Conceptual model of carbon cycling in the open waters of the Sacramento-San Joaquin Delta, detailing the major pools, rates, and drivers described in this study. The dominant organic carbon (OC) pools are macrophyte biomass and detritus, particularly those associated with invasive aquatic vegetation (IAV) such as Egeria densa. Other significant stocks include sediment carbon stocks, exogenous detritus from forest and marsh systems, phytoplankton biomass, and dissolved organic matter (OM). Ecosystem metabolism represents the conversion of inorganic carbon to OC, and vice versa, and was the focus of this study. We sought to investigate the impact of both natural metabolic drivers, including light – using turbidity as a proxy – and water temperature, and of anthropogenic metabolic drivers including wastewater treatment plant (WWTP) effluent, emergency drought barriers (EDBs) that alter flows to limit salinity intrusion during low rainfall years, and changing weather patterns in the form of wet vs. dry years. The major physical change to the OC pool is hydrologic import and export

Metabolic estimates were made in the context of a conceptual model for aquatic OC in the Delta, which is depicted in Fig. 2. The dominant OC pools in the Delta are macrophyte biomass (including detritus), phytoplankton, sediment C, and dissolved organic matter (DOM). The major abiotic process controlling the size of the OC pool is hydrologic transport, which particularly impacts phytoplankton, detritus, and DOM. This study is focused on ecosystem metabolism, which is defined here as the set of in situ biological processes that alter the size of the OC pool, and its drivers. Metabolism was calculated using a diel open-water metabolic technique that is unable to differentiate between the different pools of OC, with all metabolic estimates integrating over the entire aquatic ecosystem.

The metabolic rates utilized in this study are (a) gross primary production (GPP), which is the conversion of dissolved inorganic carbon (DIC) to OC by autotrophic photosynthesis, (b) ecosystem respiration (R), which is the transformation of OC to DIC by both autotrophs and heterotrophs, and (c) net ecosystem metabolism (NEM), which is defined as |GPP|—|R| and represents total balance of OC within an ecosystem. When calculated in units of DO, negative NEM indicates net heterotrophy and that a system is a sink of OC. Positive NEM indicates net autotrophy and that a system is a source of OC.

### Metabolic Estimates

Ecosystem metabolism was calculated using a diel open-water technique based on temporal changes in DO (Odum, 1956) following methodology detailed by Staehr et al. (2010). All calculations were made in Python. Unless otherwise stated, metabolic results are presented in units of areal molar DO per day (mmol m^−2^ d^−1^), and assume a 1-to-1 metabolic conversion between DO and C.

The governing equation of the diel technique relates changes in oxygen concentration over time ($\frac{d{O}_{2}}{dt}$) to the various sources and sinks of DO in aquatic systems (Odum, 1956; Staehr et al., 2010):

1 $$\frac{d{O}_{2}}{dt}=GPP-R-F-A$$

where F is gas transfer between the water column and the atmosphere, and A is advection, which was generally assumed to be negligible following monthly averaging (see below). F was calculated as (Staehr et al., 2010):

2 $$F={k\times (DO}_{meas}-{DO}_{sat})$$

where DO_meas_ is the measured DO value, DO_sat_ is the saturation concentration, and k is the piston velocity for air–water gas transfer. k was calculated as (Jähne et al., 1987; Staehr et al., 2010):

3 $$k={k}_{600}\times {\left(\frac{Sc}{600}\right)}^{-0.5}$$

where k_600_ is the piston velocity normalized to a Schmidt number (Sc) of 600, and is equal to (Cole & Caraco, 1998; Staehr et al., 2010):

4 $${k}_{600}[m {h}^{-1}]=\frac{2.07+0.215{U}_{10}^{ 1.7}\left[m {s}^{-1}\right]}{100}$$

with U_10_ the wind speed measured at 10 m above the surface. Sc was calculated based on water temperature (T, Celsius) (Staehr et al., 2010; Wanninkhof, 1992):

5 $$Sc=0.0476{ T}^{3}+3.7818 {T}^{2}-120.1 T+1800.6$$

Differences between consecutive 15 min DO measurements (dO_2_), along with estimates of F and the mixing depth (assumed equal to H), were used to calculate NEM for each 15 min interval (Cole et al., 2000; Staehr et al., 2010):

6 $${NEM}_{15}={dO}_{2}\times H-F$$

These NEM values were then separated into daytime and nighttime values based on PAR, with nighttime values defined as 15 min intervals with PAR < 1 mmol m^−2^ 15 min^−1^. Daily NEM was defined as the mean NEM_15_ for each 24 h day:

7 $$NEM=mean({NEM}_{15})$$

while R was equal to mean NEM_15_ estimated at night:

8 $$R=mean({NEM}_{15night})$$

GPP was calculated as the difference between mean NEM during daylight hours and the fraction of R that occurred during daylight hours:

9 $$GPP={NEM}_{day}-{(R}_{diel}\times \frac{{h}_{day}}{24}):$$

Diel metabolism was first calculated at daily resolution, which is the smallest possible timescale allowed by Eq. 7–9. Daily values were then averaged by month to account for advection. To evaluate seasonal metabolic status (net autotrophy vs. net heterotrophy), NEM values for each site were grouped by month across all years (2014–2025), before 1 sample t-tests were performed to assess differences from 0.

Metabolism estimates integrate over a large measurement “footprint”, that depending on flow velocity can extend as far as several km upstream of the sensor (Koopmans & Berg, 2015; Reichert et al., 2009). Several of the sensor footprints in this study encompass a heterogeneous bottom, such as a mix of bare sediments and vegetation, or of shallow and deeper water, each with a different metabolic regime impacting the production or consumption of DO. Over short timescales and when using a single sensor, this heterogeneity in benthic cover in a flowing system violates the assumption of zero advection in Eq. 1, as upstream production or consumption of DO will be recorded at the measurement location at a later time than it occurred. In our study, we applied longer-term monthly averaging of daily values to smear the advective impacts of a heterogeneous bottom on $\frac{d{O}_{2}}{dt}$ across a 24-h day, which diminishes the bias of spatial heterogeneity (Loken et al., 2021; Staehr et al., 2010). While longer-term averaging removes advective bias from metabolic estimates, it does not change the fundamental fact that these estimates integrate over a large benthic surface area. As shown below, the presence and location of this measurement footprint is important when interpreting our metabolic results.

### Analysis of Metabolic Drivers

The Delta has been substantially altered from its natural state, and is subject to numerous anthropogenic and natural stressors. Each of the stressors examined in our study is either the direct result of management action, or else is a topic of management concern.

#### Wastewater Treatment Plant (WWTP) Effluent

Prior to 2021, the largest point source of Delta dissolved inorganic nitrogen (DIN) was the EchoWater Resource Recovery Facility (hereafter EchoWater), previously named theSacramento Regional Wastewater Treatment Plant (Senn et al., 2020). To address impacts from the plant, major upgrades to EchoWater were completed in 2021. These upgrades gradually went online during the time period October 2020—June 2021, and shifted constituent DIN in wastewater effluent from ammonium (NH_4_^+^) to nitrate (NO_3_^−^), while decreasing total DIN outflows by ~ 85% (Mussen et al., 2024).

EchoWater discharges into the Sacramento River just downstream of FPT, such that the WGA mooring records impacts from the plant while the FPT mooring does not. To assess the impacts of the upgrades on ecosystem metabolism, we calculated the monthly mean GPP, R, and NEM at FPT and WGA, as well the difference in metabolic rates between the two sites (WGA-FPT). This difference was calculated to control for interannual variability in metabolism and phytoplankton biomass irrespective of plant upgrades. Given the relatively short distance between the two stations (29 km – Fig. 1), and their similar site characteristics (Kraus et al., 2017b), we assume that differences in metabolism and/or phytoplankton biomass are primarily attributed to effluent from EchoWater. In addition to metabolic rates, we also examined the concentrations of chl *a* and nitrate + nitrite (NO_x_) at both stations before and after EchoWater upgrades.

We applied an ordinary least squares (OLS) model to the pre- and post- upgrade differences in metabolic values between the two sites, organized by month, with an indicator variable used for the post-upgrade period. Model statistics (t, p, and standard error of the mean (SE)) were estimated using a heteroskedasticity and autocorrelation corrected (HAC) covariance matrix (Newey & West, 1987), with a lag time of 1 day. This model was chosen in lieu of a series of paired t tests in order to account for first-order (AR-1) autocorrelation likely to be present in our daily metabolic values.

#### Phytoplankton Blooms

In 2016, the central Delta was impacted by one of the largest phytoplankton blooms on record, and the largest recorded by our stations, composed of the diatom *Aulacoseira granulata* (Jungbluth et al., 2021). To quantify the impact of this bloom on ecosystem metabolism, and how it differed from baseline conditions, in addition to diel estimates of GPP we also calculated the phytoplankton-specific GPP (GPP_chl_) at the three central Delta sites affected by the event (LIB, RYE, TOL). GPP_chl_ was estimated using an empirical chl *a* parameterization as described by Jassby et al. (2002):

10 $${GPP}_{chl}= \left(4.61\times \psi \times \frac{B*I}{{k}_{d}}\right)*\raisebox{1ex}{$1$}\!\left/ \!\raisebox{-1ex}{$12$}\right.$$

where B is chl *a* concentration [mg m^−3^], I is surface irradiance [E m^−2^ d^−1^], ψ is an efficiency factor equal to 0.728 mg C [mg chl *a*]^−1^ [E m^−2^]^−1^, and the $$\raisebox{1ex}{$1$}\!\left/ \!\raisebox{-1ex}{$12$}\right.$$ scaling represents a stoichiometric conversion from mg C to mmol O_2_ assuming a 1 to 1 molar ratio between C and O_2_. k_d_ is the light extinction coefficient [m^−1^], and was calculated using a site-specific calibration based on turbidity (Tu) [FNU] recorded by the moorings:

11 $${k}_{d}=0.077\times Tu+1.05$$

Further explanation of constants is detailed within Jassby et al. (2002). 15 min values of GPP_chl_ were averaged first by day, and then by month, for better comparison with DO-based GPP estimates. As a proxy for total phytoplankton throughout the Delta, we also calculated the daily mean chl *a* concentration at each site.

#### Emergency Drought Barrier (EDB)

Water year (WY) 2021 was the driest since 1977 for the state of California (Bouma-Gregson et al., 2024), resulting in the deployment of an EDB in the central Delta to prevent saltwater intrusion. This barrier was placed across the west False River, separating the heavily vegetated backwater Franks Tract from the San Joaquin River (Hartman et al., 2022), near the location of the JER mooring. The EDB was deployed from June 2021-October 2022. By cutting the impact of this vegetation off from the footprint of the JER sensor (Fig. 1), the installation of the barrier is likely to impact ecosystem metabolism estimated at this location.

To investigate impacts of the EDB we compared the mean metabolism by month during the time period when the EDB was deployed vs. the mean monthly metabolism during the 12 months immediately prior to EDB deployment and the 14 months immediately following its removal. To control for possible environmental differences between these time periods we made the same comparison at LIB, which like JER, is heavily vegetated but was not impacted by an EDB. We also calculated the difference in metabolism between periods of EDB presence and absence by month at both locations. At JER we applied the same OLS model used to assess WWTP upgrades ("Wastewater Treatment Plant (WWTP) Effluent" section) to daily metabolic rates between the periods during and immediately outside of the period of EDB deployment, organized by month, with an indicator variable used to identify the EDB deployment. As with WWTP upgrades, model statistics (t, p, and SE) were estimated using a HAC covariance matrix with a lag time of 1 day to account for AR-1 autocorrelation.

#### Physical Drivers

We tested the impact of temperature (T), Tu, and seasonality on GPP and R using a multivariate linear mixed effect modeling (LMM) approach. Due to a wide range of values, Tu was log_10_ transformed. Seasonal impacts were assessed using a sin and cos transformation of the integer month (mo). We used the MixedLM.from_formula function with a Limited-memory Broyden-Fletcher-Goldfarb-Shanno (LBFGS) optimizer via the Python statsmodels.api package. The final LMM was:

12 $$R,GPP\sim T+{log}_{10}\left(Tu\right)+{mo}_{sin}+{mo}_{cos};Re=1+T+{log}_{10}(Tu)$$

where ${mo}_{sin}=\mathrm{sin}(2\pi *mo/12)$ and ${mo}_{cos}=\mathrm{cos}(2\pi *mo/12)$. This formulation allows for random effects (Re) for the intercept, T, and Tu coefficients based on site, but does not vary seasonally across sites.

To evaluate the impacts of wet vs. dry years, and flow through the DCC (Q_DCC_), we calculated integrated rates of R, GPP, and NEM as the sum of daily diel metabolism values for each WY, which spans Oct 1—Sep 30. Due to the presence of the EDB, WY21 and WY22 were excluded from JER for this analysis. To better present net C burial or release, these estimates are presented in terms of g C y^−1^, which we calculated assuming a 1 to 1 molar relationship between DO and C. Discharge measured by FPT (Q_FPT_) in the Sacramento River was used as a proxy for Delta water availability. We fit the following LMM, also using the LBFGS optimizer, with site-specific random intercepts and coefficients for Q_FPT_ and Q_DCC_:

13 $${R}_{WY},{GPP}_{WY}\sim {Q}_{FPT}+{Q}_{DCC}; Re=1+{Q}_{FPT}+{Q}_{DCC}$$

where R_WY_ and GPP_WY_ are the sum total R and GPP for each WY.

## Results

### General Metabolic Trends

**Fig. 3:**
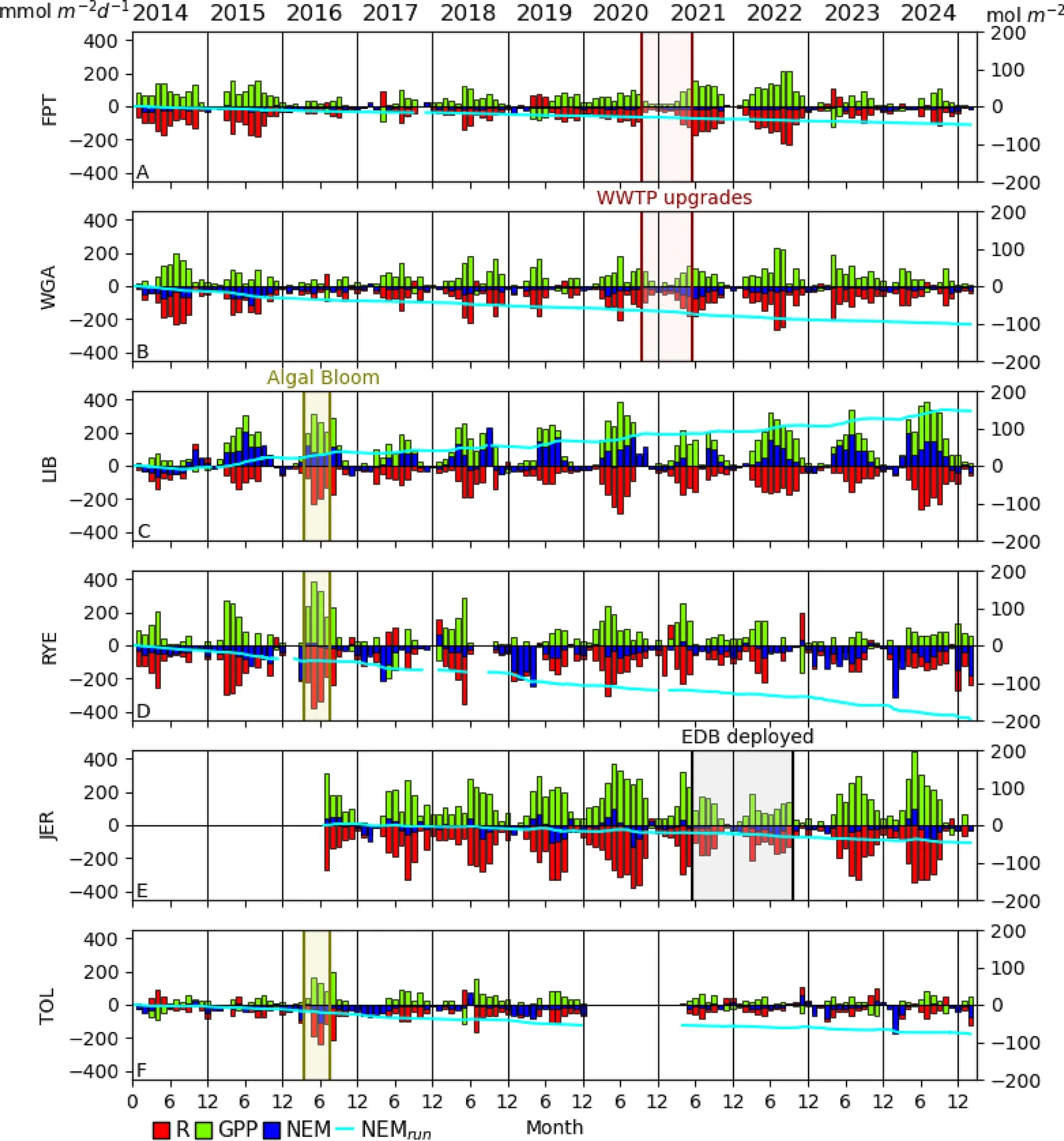
Monthly metabolism results – respiration (R), gross primary production (GPP), and net ecosystem metabolism (NEM)—at the six sites, along with running NEM (NEM_run_-right y axis) over the study period. There was a strong seasonal signal at all sites, with enhanced metabolic rates in the summer, as well as substantial interannual variability linked with precipitation, discharge, and management actions. All sites other than LIB were net heterotrophic. Also shown are the timings of three key events that were examined in this study: upgrades to a wastewater treatment plant (WWTP) located in the Sacramento River between FPT and WGA that were completed in 2021, a major algal bloom in the central Delta from Apr-Jul 2016, and deployment of an emergency drought barrier (EDB) near JER from Jun 2021- Oct 2022

Clear seasonal signals were evident in metabolism results at all sites, with minimum R and GPP in the winter and maximum values in the spring and/or summer (Fig. 3). Values of R and GPP were greatest at the highly vegetated LIB and JER locations, with R and GPP at JER as high as −400 and 450 mmol m^−2^ d^−1^, respectively. There was substantial interannual metabolic variability at all sites, including impacts from WWTP upgrades at WGA, the *A. granulata* algal bloom at LIB, RYE, and TOL, and the EDB at JER (Fig. 3).

**Fig. 4:**
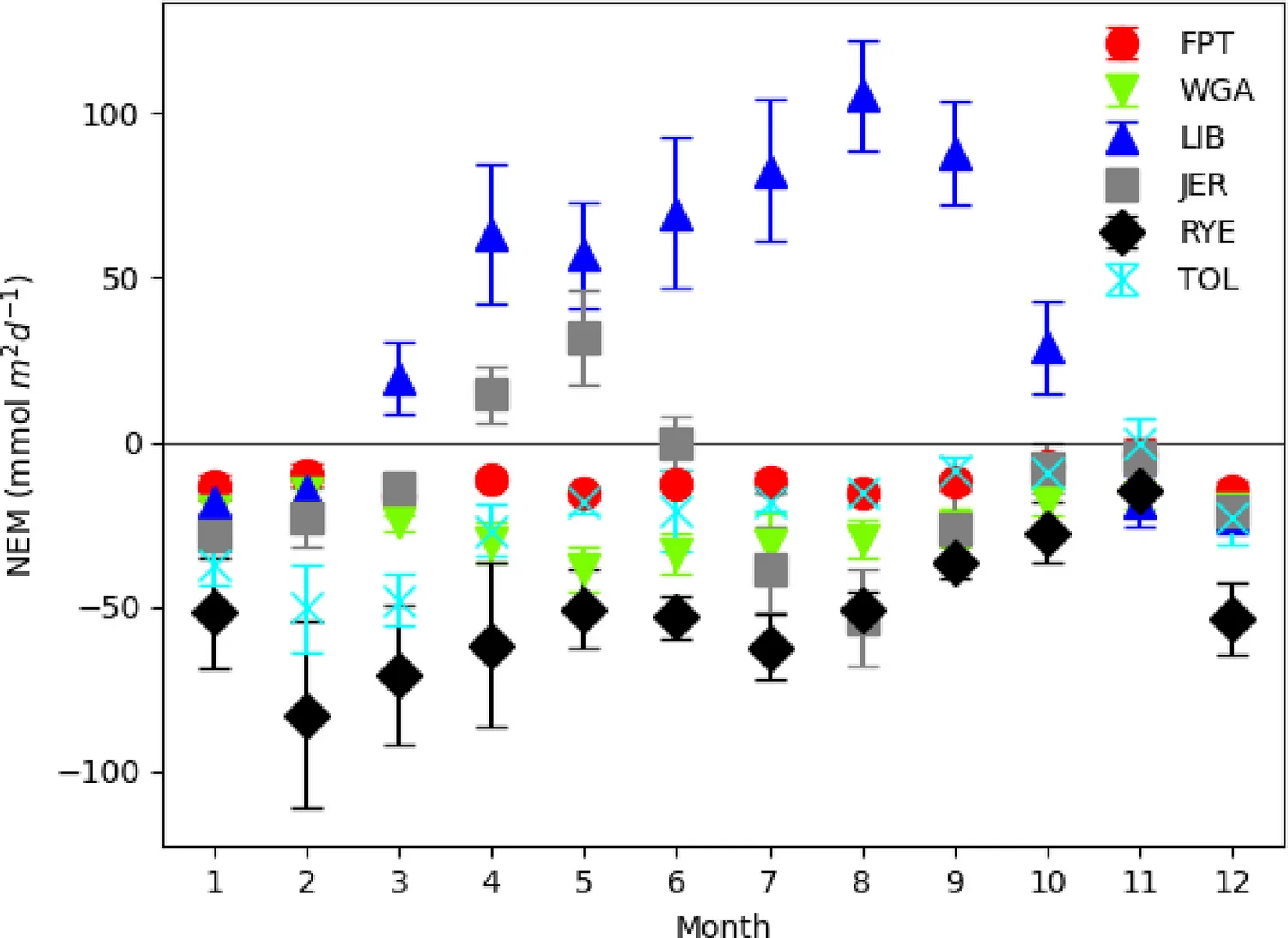
Summary of mean net ecosystem metabolism (NEM) by month at each site, +- SE. Most sites tended to be net heterotrophic (NEM < 0) throughout the year, with the exception of LIB, which was autotrophic, and JER, which was nearly balanced. Sites in the Sacramento River (FPT, WGA) are presumed to be sustained by allochthonous C, as there are no clear in situ sources of C at these locations. RYE and TOL, which are downstream of LIB, were most strongly heterotrophic, which we attribute to remineralization of C produced at LIB and other heavily vegetated sections of the central Delta

Mean monthly NEM was less than 0 – indicating net heterotrophy – at all sites for all months with the exception of LIB (March—October) and JPT (March—April), which were autotrophic (Fig. 4). Mean NEM was significantly different from 0 (*p* < 0.01) at all sites, with LIB demonstrating net autotrophy and the rest net heterotrophy (Table 1).

**Table 1:** Results from 1-sample t tests on difference from 0 for monthly net ecosystem metabolism (NEM) values. LIB was net autotrophic (NEM > 0), while all other sites were net heterotrophic (NEM < 0)

Site	NEM	SE	n	t	p
FPT	−12.0	0.9	133	−13.7	< 0.001*
WGA	−25.2	1.6	134	−15.3	< 0.001*
LIB	36.0	5.8	134	6.2	< 0.001*
RYE	−51.4	4.9	127	−10.5	< 0.001*
JER	−15.0	3.8	104	−4.0	< 0.001*
TOL	−22.7	2.7	119	−8.5	< 0.001*

Bold* indicates *p* < 0.05

### WWTP Upgrades

**Fig. 5:**
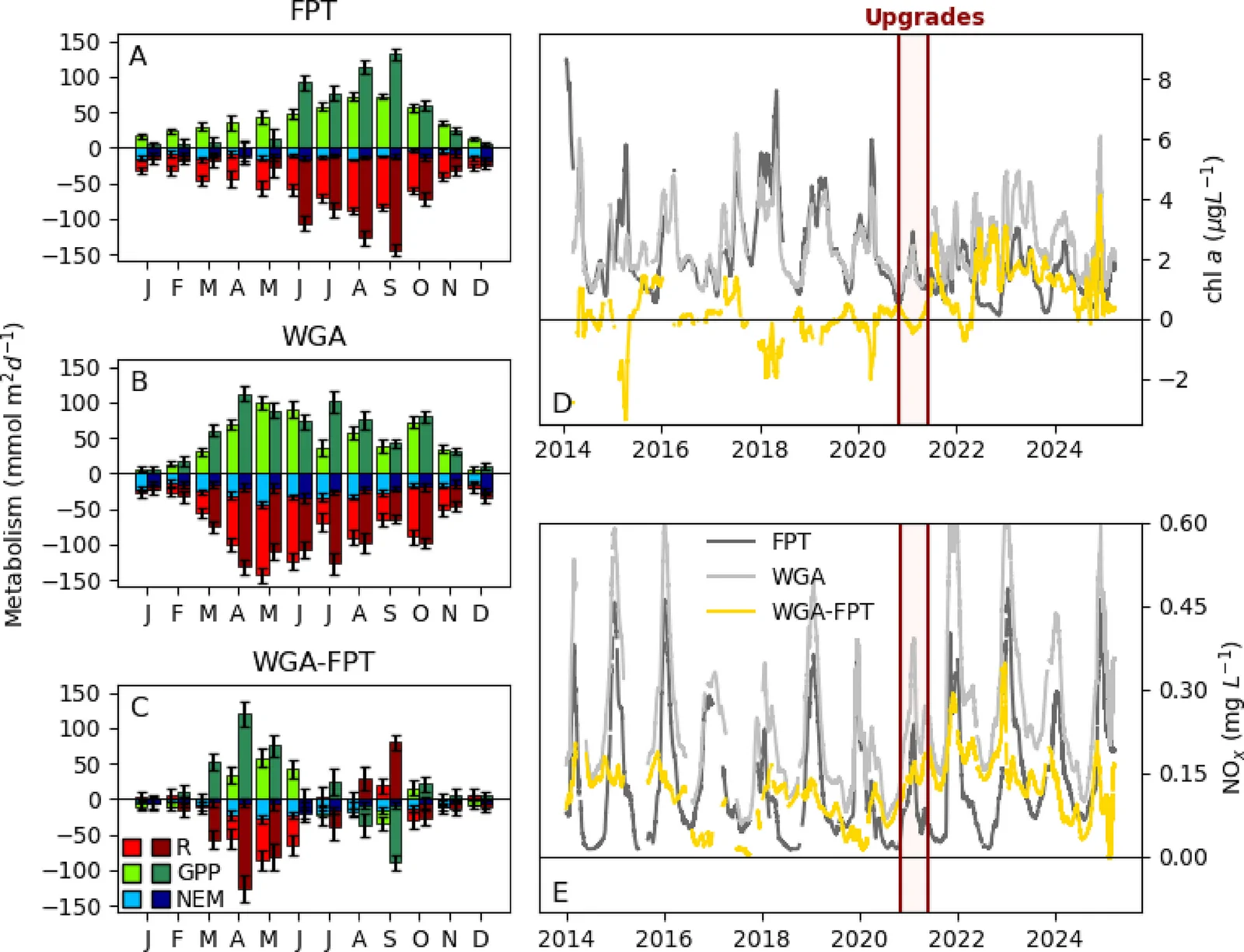
Mean monthly respiration (R), gross primary production (GPP) and net ecosystem metabolism (NEM) at (**A**) Freeport (FPT), (**B**) Walnut Grove (WGA), and (**C**) the difference between them (WGA – FPT). Light and dark colors indicate, pre- and post—EchoWater upgrades, respectively, with error ranges defined as ± SE from the OLS model. (**D**) and (**E**) depict 30 day running averages of chl a and NO_x_ concentration, with the yellow line representing WGA-FPT. FPT is located within the Sacramento River upstream of EchoWater and acted as our control site, while WGA is located ~ 30 km downstream and is the site most heavily impacted by EchoWater effluent

FPT and WGA exhibited slightly different seasonal metabolic patterns, with values peaking in the late summer/fall at FPT vs. the spring at WGA (Fig. 5A and B). Following upgrades, both R and GPP fell at FPT during Jan-May (Fig. 5A), while staying relatively constant at WGA (Fig. 5B). As a result, the difference in R and GPP between WGA and FPT for Jan-May tended to be greater following the upgrades than before (Fig. 5C), with this effect particularly strong during March and April (Table 2).

**Table 2:** Summary of OLS model statistics assessing changes in the difference (WGA-FPT) between daily respiration (R), gross primary production (GPP), and net ecosystem metabolism (NEM) pre- and post- EchoWater upgrades

	ΔR		ΔGPP		ΔNEM	
Month	t	p	t	p	t	p
Jan	−1.20	0.231	1.44	0.150	0.24	0.812
Feb	−2.13	0.033*	2.21	0.027*	0.06	0.956
Mar	−3.31	0.001*	3.66	< 0.001*	1.24	0.214
Apr	−2.60	0.009*	3.14	0.002*	2.33	0.020*
May	0.19	0.849	0.78	0.437	2.31	0.021*
Jun	3.97	< 0.001*	−3.50	< 0.001*	0.33	0.743
Jul	−2.76	0.006*	3.13	0.002*	0.87	0.386
Aug	1.84	0.066	−1.43	0.154	1.44	0.150
Sep	4.11	< 0.001*	−3.72	< 0.001*	1.09	0.277
Oct	0.25	0.803	0.22	0.825	1.27	0.205
Nov	0.02	0.988	0.19	0.850	0.37	0.711
Dec	−2.11	0.035*	2.00	0.045*	−1.14	0.254

Bold* indicates *p* < 0.05

Following EchoWater upgrades, chl *a* concentrations were approximately 2 µg L^−1^ greater at WGA vs. FPT, after being approximately equal at the two sites before. As with metabolic values, this change was driven by decreases at in chl *a* at FPT rather than increases at WGA (Fig. 5D). Although the dominant DIN species in EchoWater effluent changed from NH_4_^+^ to NO_x_ following upgrades, differences in NO_x_ between the WGA and FPT were similar before and after upgrades (Fig. 5E), suggesting substantial historical nitrification of NH_4_^+^ discharged by the plant (Kraus et al., 2017b).

### Phytoplankton

**Fig. 6:**
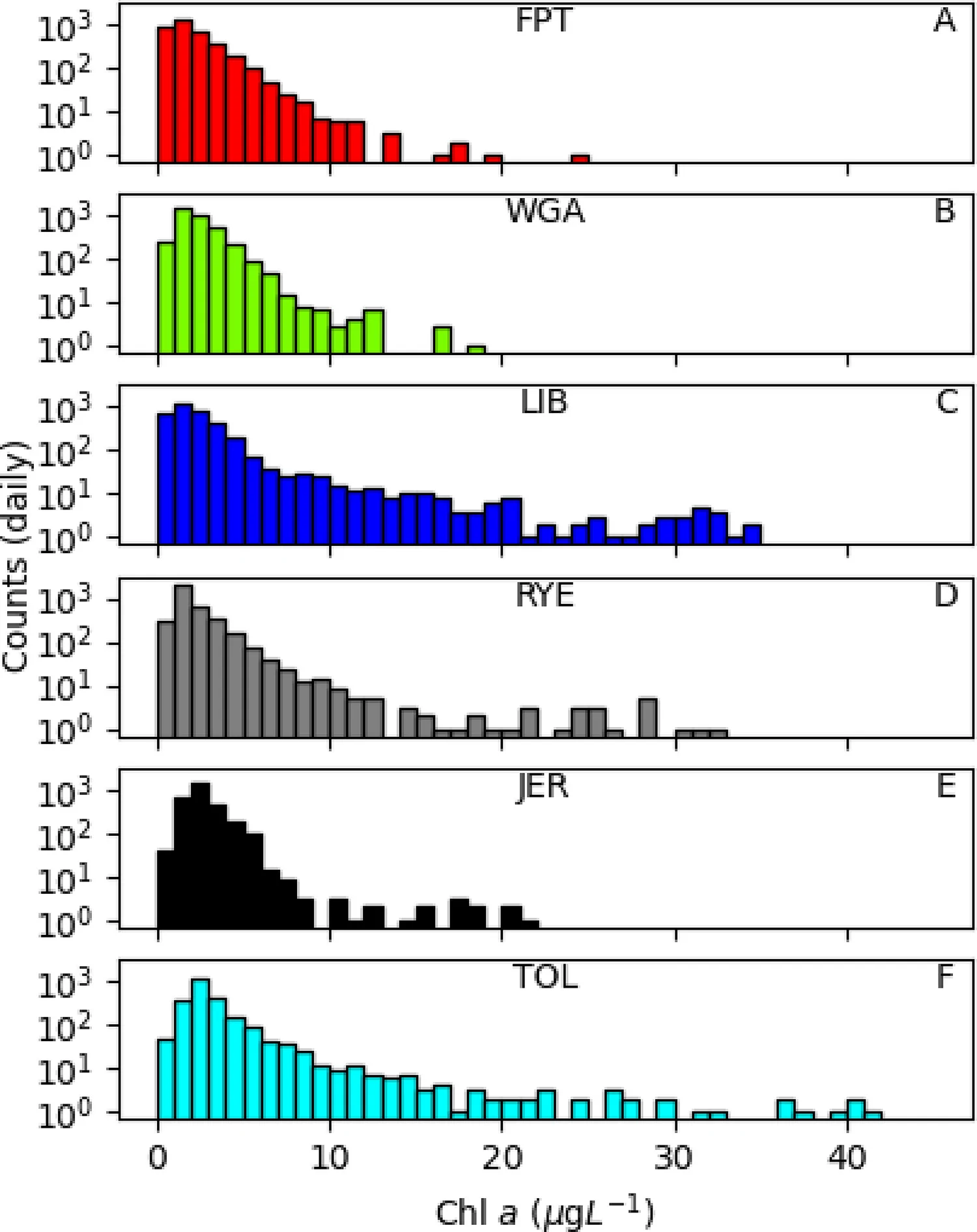
Histograms of mean daily chl a at the 6 sites in this study. Chl a concentrations are very low in the Delta, consistently < 10 µg L^−1^ (please note the log_10_ scale for number of counts). As a result, gross primary production (GPP) is attributed primarily to photosynthesis by macrophytes and benthic microalgae, rather than phytoplankton

**Fig. 7:**
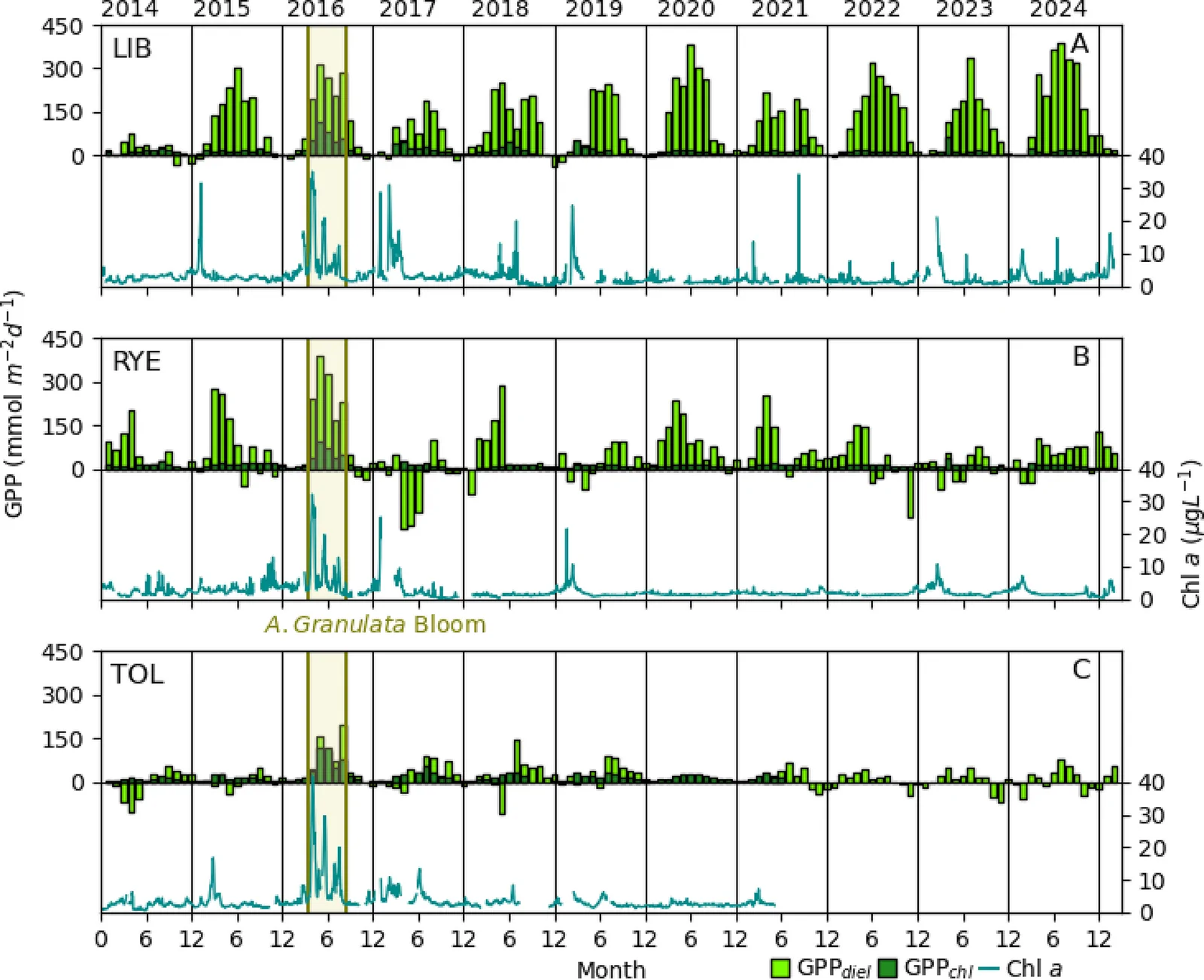
Summary of the contribution of phytoplankton to gross primary production (GPP) measured at the three sites (LIB, RYE, and TOL) that were impacted by the 2016 bloom of the diatom Aulacoseira granulate. GPP attributed to phytoplankton (GPP_chl_) generally tended to be much smaller than total GPP (GPP_diel_), with the exception of TOL. Please note TOL does not have chl a data or GPP_chl_ estimates following June 2021. Teal lines represent daily mean chl a

Phytoplankton levels tended to be very low throughout the Delta, with ~ 90% of daily mean chl *a* measurements < 5 µg L^−1^ at all sites (Fig. 6). Accordingly, GPP_chl_ at the 3 central Delta sites was low, with baseline estimates of GPP_chl_ < 50 mmol m^−2^d^−1^ (Fig. 7). During the 2016 *A. granulata* bloom both chl *a* and GPP_chl_ substantially increased, with daily mean chl *a* as high as 42 µg L^−1^ at TOL, and monthly GPP_chl_ as high as ~ 120 mmol m^−2^d^−1^ at both LIB and TOL (Fig. 7). Despite substantial productivity from the bloom, GPP_chl_ remained < 50% of GPP_diel_ at both RYE and LIB. However, GPP_chl_ was of similar magnitude to GPP_diel_ at TOL, both during the bloom and during baseline conditions, suggesting phytoplankton compromise the bulk of primary production at this location.

### Emergency Drought Barrier (EDB)

**Fig. 8:**
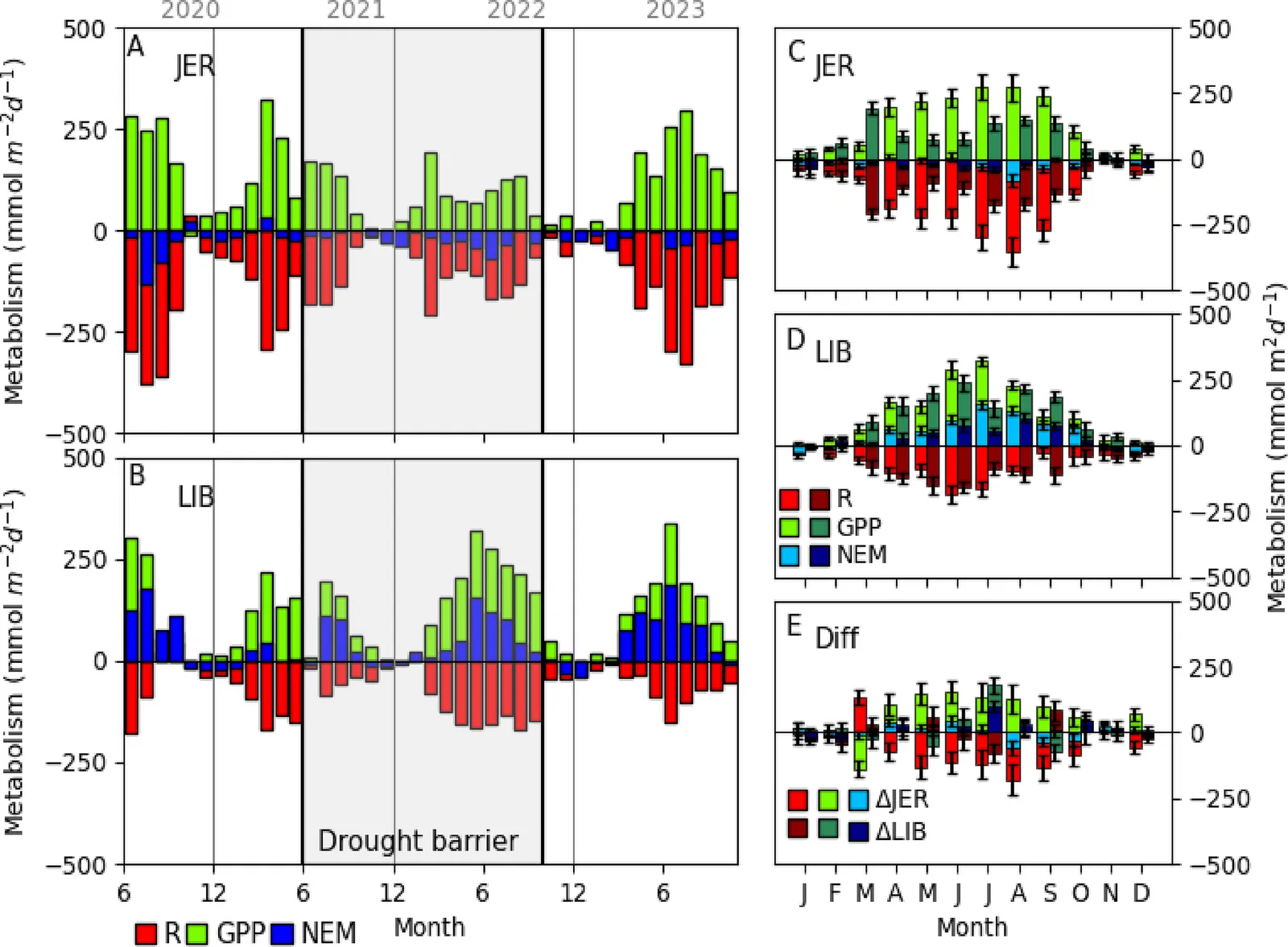
Impact of the deployment of an emergency drought barrier (EDB) on (A) respiration (R), gross primary production (GPP) and net ecosystem metabolism (NEM) at JER, with (B) LIB, which was unaffected by the EDB, shown as a control. (C) and (D) depict the mean metabolism by month of the 12 months both prior and 14 months following the drought EDB (light bars) vs. mean metabolism with EDB installed (dark bars), with (E) showing difference between them. Error ranges are defined as ± SE from the OLS model. The EDB was installed from June 2021 – October 2022 to prevent salt water intrusion into the upper Delta during a historic low water year. This barrier cut off connection between the JER mooring and the heavily vegetated Franks Tract, resulting in substantially lower metabolic values during this time period that were not mirrored at the LIB control site

Monthly values of R and GPP at JER primarily decreased during the period of EDB deployment (Table 3), due to the loss of connectivity of the JER mooring with the heavily vegetated Franks Tract. Magnitudes of R and GPP fell by as much as 150–180 mmol m^−2^ d^−1^ depending on month, with these same decreases in metabolism not observed at LIB (Fig. 8). Overall, mean rates of R and GPP at JER fell by 40–70% during the April – Oct period of peak productivity.

**Table 3:** Summary of OLS model statistics assessing changes between daily respiration (R), gross primary production (GPP), and net ecosystem metabolism (NEM) at JER during the period of EDB deployment and the 12 months prior to its installation and the 14 months immediately following its removal

	ΔR		ΔGPP		ΔNEM	
Month	t	p	t	p	t	p
Jan	0.14	0.885	0.18	0.857	−0.63	0.532
Feb	−0.50	0.617	1.02	0.306	0.84	0.403
Mar	−4.76	< 0.001*	4.63	< 0.001*	0.72	0.469
Apr	1.93	0.054	−2.67	0.008*	−2.79	0.005*
May	2.95	0.003*	−3.82	< 0.001*	−1.23	0.218
Jun	2.65	0.008*	−3.81	< 0.001*	−1.91	0.056
Jul	2.23	0.026*	−2.44	0.015*	−0.72	0.472
Aug	3.05	0.002*	−2.26	0.024*	2.09	0.036*
Sep	2.83	0.005*	−2.36	0.018*	2.12	0.034*
Oct	2.45	0.014*	−1.76	0.079	1.23	0.220
Nov	−0.16	0.873	−0.72	0.469	−1.56	0.120
Dec	2.32	0.02*	−2.70	0.007*	−0.77	0.442

Bold* indicates *p* < 0.05

### Physical Drivers

**Fig. 9:**
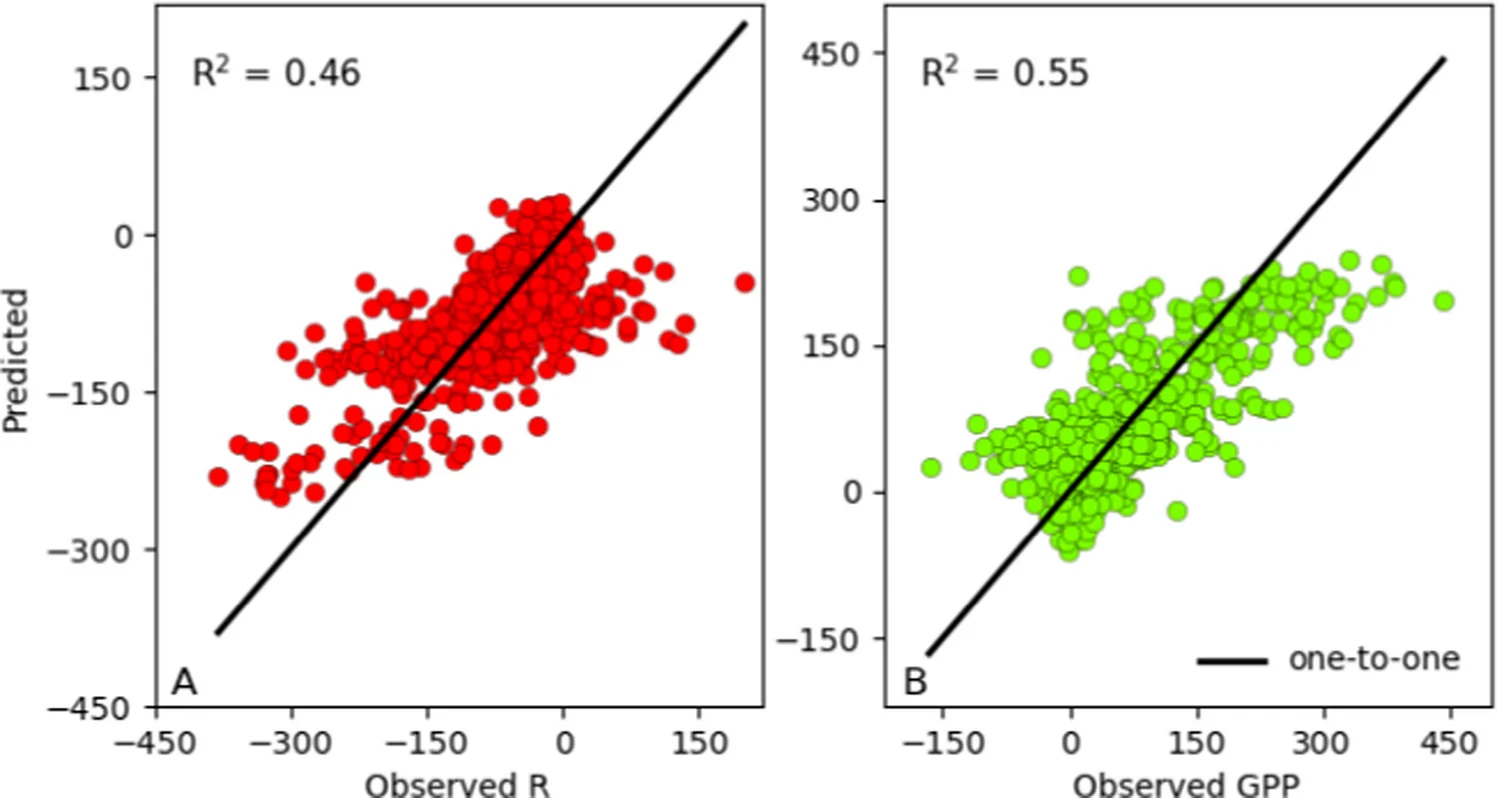
Predicted vs observed values from linear mixed effect model (LMM) results for monthly respiration (R) (**A**) and gross primary production (GPP) (**B**) plotted alongside a one-to-one line. LMMs for both metabolic rates incorporated T, Tu, and a seasonal dummy variable, and allowed for site-specific random effects. Both models explained roughly ~ 50% of the variance seen in R and GPP as shown by R^2^ values. The LMMs tended to under-predict higher metabolic rates, as evidenced by deviations of predicted values to the left of the one-to-one line for high (negative) values of observed R, and deviations to the right of the one-to-one line for high values of observed GPP

Our LMM demonstrated strong seasonal signals in both GPP and R models, as mon_sin_ had fixed effect (present across all sites) coefficients of −36.4 and 32.5 (*p* < 0.01), respectively. T and Tu were confirmed as physical drivers of GPP, while only T was a driver of R. The fixed effect T coefficients for R and GPP were −10.1 (*p* < 0.01) and 7.8 (p = 0.01), respectively, indicating increasing R and GPP with T. Fixed effect log_10_(Tu) coefficients were −73.4 (*p* < 0.01) for GPP, indicating decreased GPP with increasing Tu, and 28.3 (p = 0.21) for R, indicating uncertain impacts. Correlation coefficients (R^2^) for the R and GPP models were 0.46 and 0.55 (Fig. 9), respectively, suggesting that seasonality, T, and Tu explain roughly half of the variance for both R and GPP. Fixed effect coefficients are summarized in Table 4, while site-specific, random effects coefficients are presented in Table S1.

**Table 4:** Fixed effect coefficients and statistics for linear mixed effect models (LMMs) for monthly respiration (R) and gross primary production (GPP) [mmol O_2_ m^−2^ d^−1^] based on temperature (T), turbidity (Tu), and seasonality (mon_sin_ and mon_cos_). Please note that R is negative and GPP is positive, so negative coefficients indicate enhanced R and vice versa. Lower and upper bounds are 2.5 and 97.5 percentile values, respectively

Model	variable	value	SE	p	lower	upper
R	Intercept	60.4	36.0	0.09	−10	131
R	T	−10.0	2.3	< 0.001*	−14.5	−5.6
R	log_10_(Tu)	28.3	22.7	0.21	−16.2	72.9
R	^mo^sin	−36.4	8.0	< 0.001*	−52.2	−20.7
R	^mo^cos	−12.7	10.5	0.23	−33.2	7.8
GPP	Intercept	3.3	40.8	0.94	−76.7	83.3
GPP	T	7.9	3.0	0.009*	2.0	13.8
GPP	log_10_(Tu)	−72.0	13.4	< 0.001*	−98.2	−45.7
GPP	^mo^sin	32.5	8.2	< 0.001*	16.5	48.4
GPP	^mo^cos	−1.9	10.6	0.86	−22.6	18.8

Bold* indicates *p* < 0.05

**Fig. 10:**
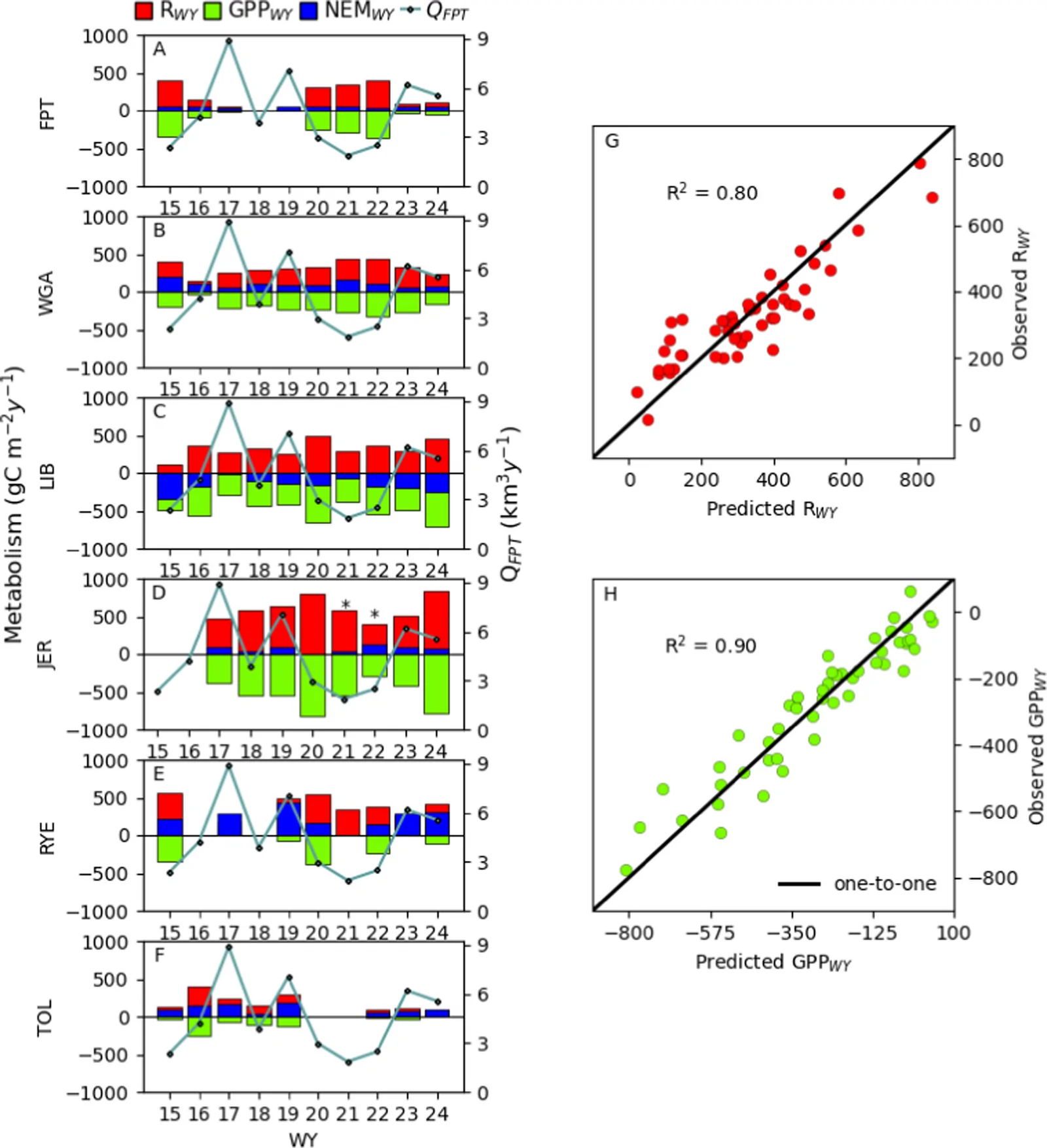
Annual estimates of carbon metabolism averaged by water year (WY), including respiration (R_WY_), gross primary production (GPP_WY_) and net ecosystem metabolism (NEM_WY_) (A-F), along with total discharge recorded at FPT (Q_FPT_) which was used as a proxy for water availability. (G,H) Results from linear mixed effect models (LMMs) for R_WY_ and GPP_WY_ vs. Q_FPT_ and discharge through the Delta Cross Channel (Q_DCC_). Q_FPT_ had negative impacts on the magnitudes of R_WY_ (p = 0.01) and GPP_WY_ (p = 0.01), indicating that wetter WYs had lower areal metabolism than dryer WYs, while Q_DCC_ had weaker, nonsignficant positive impacts (p = 0.15 and p = 0.20, respectively). Please note that as these are in units of C, the signs for GPP_WY_ and R_WY_ are reversed relative to monthly GPP and R estimates in units of DO

R_WY_ and GPP_WY_ showed substantial variation across sites and WY, with magnitudes of R_WY_ and GPP_WY_ ranging from near zero for some WY at TOL and FPT to 800 g C m^−2^ y^−1^ at JER in WY20 and WY24 (Fig. 10A-F). NEM_WY_ mirrored our monthly results, as all sites except LIB tended to be heterotrophic, with LIB demonstrating consistent net autotrophy. Please note that as WY results are in units of C, the signs for R_WY_, GPP_WY_ and NEM_WY_ are reversed relative to monthly estimates in units of DO.

Variance of R_WY_ and GPP_WY_ were well explained by our LMM, with R^2^ = 0.80 and 0.90, respectively (Fig. 10GH). Q_FPT_, which was used as a proxy for precipitation, had fixed effect coefficients of −27 (p = 0.01) and 36 (*p* = 0.01) for R_WY_ and GPP_WY_, respectively, indicating lower metabolism during wetter years (Table 5). Q_DCC_ had less certain positive effects on the magnitudes of R_WY_ and GPP_WY_ (p = 0.15 and 0.20, respectively; Table 5). These impacts were driven primarily by JER, which had combined random and fixed effect Q_DCC_ coefficients of 340 and −440 for R_WY_ and GPP_WY_, nearly three times larger than the combined effects of Q_DCC_ at any other site (Table 5, Table S2).

**Table 5:** Fixed effect coefficients and statistics for linear mixed effect models (LMMs) for respiration and gross primary production by water year (R_WY_ and GPP_WY_, respectively) [g C m^−2^ y^−1^] based on discharge at the FPT mooring (Q_FPT_) and discharge through the Delta Cross Channel (Q_DCC_). Please note units of C, such that R_WY_ is positive and GPP_WY_ is negative, so positive coefficients indicate enhanced R_WY_ and vice versa. Lower and upper bounds are 2.5 and 97.5 percentile values, respectively

Model	variable	value	SE	p	lower	upper
^R^WY	Intercept	332	97.7	0.001*	141	524
^R^WY	^Q^FPT	−26.9	10.5	0.010*	−47.5	−6.4
^R^WY	^Q^DCC	119	83.0	0.153	−44.1	282
GPP_WY_	Intercept	−286	100	0.004*	−483	−89.8
GPP_WY_	^Q^FPT	35.6	13.2	0.007*	9.8	61.4

Bold* indicates *p* < 0.05

## Discussion

### Delta C Cycling

Our results suggest a nuanced view of C utilization in the Delta defined by regions of both net autotrophy and net heterotrophy (Table 1, Fig. 4), in contrast to previous studies that have suggested that the system is primarily heterotrophic and relies on subsidies of allochthonous C supplied through freshwater runoff (Sobczak et al., 2005). Sites in the Sacramento River (FPT, WGA) were heterotrophic with no obvious autochthonous sources, suggesting that these riverine regions within the Delta rely on inputs of allochthonous C as suggested by Sobczak et al. (2005). Conversely, LIB – located at the outflow of the heavily vegetated Cache Slough complex – was strongly autotrophic, suggestive of it being a source of autochthonous C to other regions of the Delta. RYE is located directly downstream of LIB (Fig. 1), and displayed the strongest net heterotrophy of any site (Fig. 4, Table 1), likely due to aerobic decomposition of autochthonous C exported from LIB. TOL – another deep-water location in the central Delta – also demonstrated strong net heterotrophy. Taken together, these results suggest that the larger channels in the Delta remineralize autochthonous C produced in shallower, more heavily-vegetated regions of the system.

Our results agree with a growing body of research suggesting that much of the Delta is transitioning from a turbid system dominated by pelagic C and nutrient cycling to a clear-water one dominated by benthic processes. The past two decades have seen the rapid proliferation of invasive aquatic vegetation (IAV) in the Delta, including both submersed and floating aquatic vegetation (SAV and FAV, respectively) (Cooke et al., 2018). Consequently, aquatic macrophytes are now the largest contributor to both primary production (Boyer et al., 2023) and DIN uptake (Gross et al., 2024) in the region. The most prolific SAV in the Delta is Brazilian waterweed (*Egeria densa*), and the two primary FAV are water hyacinth (*Pontederia* *crassipes*) and water primrose (*Ludwigia* spp.) (Cooke et al., 2018; Khanna et al., 2018). Recent years have seen a particular increase in IAV coverage within the central Delta near LIB, including a four-fold expansion in areal extent of *Ludwigia* spp. during the period 2004–2016 (Khanna et al., 2018). Mean monthly NEM at LIB was 36 mmol m^−2^ d^−1^ (Table 1), similar in magnitude to values reported for a rapidly growing seagrass meadow (Berger et al., 2020). Mean NEM_WY_ was −170 g C m^−2^ y^−1^ (Fig. 10), within the range of literature values for *E. densa* reported by Boyer et al. (2023). The transition from pelagic to benthic processes is often attributed to a positive feedback loop whereby low turbidity leads to higher light levels encouraging SAV and FAV growth, which in turn diminishes sediment resuspension through increased flow attenuation, which encourages further macrophyte growth and so on (Hansen & Reidenbach, 2012; Krause-Jensen et al., 2012). This feedback loop likely explains the shift in production towards SAV and FAV in the Delta, as expansion of IAV in the Delta is associated with decreases in water column turbidity (Hestir et al., 2016; Lacy et al., 2021), which in turn is linked to increased IAV growth (Khanna et al., 2018) and concomitant increases in metabolism.

One outcome of this shift in metabolism towards IAV is that phytoplankton are a minority source of autochthonous C within the Delta. Phytoplankton biomass in our study was low, with more than 90% of daily mean chl *a* values < 5 µg L^−1^ across all sites (Fig. 6). The ratio of GPP from phytoplankton (GPP_chl_) to total GPP (GPP_diel_) defines the proportion of primary production that can be attributed to phytoplankton activity. For example, a ratio of 0.5 would suggest equal contributions between phytoplankton and all other primary producers in the Delta. The GPP_chl_: GPP_diel_ ratios across the three central Delta sites were 0.14, 0.24, and 0.93 at LIB, RYE, and TOL, respectively, indicating minor contributions from phytoplankton to primary production at LIB and RYE, but that production at TOL is primarily driven by phytoplankton. For comparison, the ratio of production of phytoplankton to macrophytes across the entire Delta was previously reported as 0.35–0.5 (Cloern et al., 2021). As LIB is the most heavily vegetated site in our study, and RYE is often impacted by production near LIB, these sites fell below this range. TOL, in contrast, is a deep-water site far from shallow, vegetated areas. As a result, it had the lowest GPP of any of the 6 sites in our study (Fig. 3), helping explain the comparatively high contributions from phytoplankton.

The Delta has low planktonic productivity despite high nutrient levels above those associated with deleterious impacts in similar systems, such as the Chesapeake Bay (Elser et al., 2007; Paerl, 2009). Typical values of GPP_chl_ across the three central Delta sites were < 20 mmol m^−2^ d^−1^ (Fig. 7), on the lower end of global pelagic GPP reported for estuaries (~ 2–250 mmol m^−2^ d^−1^; Cloern et al., 2014) and consistent with past estimates of planktonic GPP in the lower salinity regions of SFE (Cloern et al., 2017). To date, high turbidity, particularly in non-vegetated regions, and filtering by invasive clams including *Corbicula fluminea* (Lopez et al., 2006) have mitigated classic eutrophic patterns of phytoplankton blooms and subsequent hypoxia in the Delta (Cloern, 2001; Kimmerer & Thompson, 2014), although a major HAB event in the lower, saline reaches of the SFE in 2022 (Nardelli et al., 2026) suggests that this paradigm may be changing. Phytoplankton blooms in the lotic portions of the Delta are rare, although cyanobacterial HABs in quiescent backwaters are a major concern (Bouma-Gregson et al., 2024). Our 11-year monitoring record captured only one major bloom – an overgrowth of the diatom *A. granulata* that persisted approximately 4 months (April-July) in 2016 in the central and lower Delta. During this bloom, GPP_chl_ increased by ~ 5 × at LIB, RYE, and TOL, although at LIB and RYE GPP_chl_ still accounted for < 50% of total GPP (Fig. 7). Impacts of the bloom were more substantial at TOL, where phytoplankton productivity accounted for > 90% of GPP (Fig. 7C). The large respiratory demand and subsequent bloom collapse precipitated record R and strongly negative NEM at TOL during June–August 2016 (Fig. 3F), likely to due to aerobic decomposition of phytoplankton detritus.

### Ecosystem Metabolism from High-frequency Water Quality Monitoring Arrays

The Sacramento-San Joaquin Delta is one of the most closely monitored estuarine systems in the world. Accurate estimates of metabolic rates are important for resource managers to understand the health of the Delta and the impact of management actions. While past measurements of aquatic Delta metabolism have primarily utilized the chl *a* approach (Eq. 10 – e.g. Cloern et al., 2017), there have been limited studies of Delta metabolism based on DO, with only one to our knowledge presenting values in areal units accounting for water column depth (i.e. m^−2^—Loken et al., 2021). This previous work was limited to the Sacramento River Deep Water Ship Channel – a heavily managed, artificial channel used for ship transport not sampled in our study – and only encompassed 3 months of data collection (July–September 2019). Nevertheless, Loken et al. (2021) reported similar values to our results with estimates of GPP and R of 150 and −200 mmol m^−2^ d^−1^, respectively, within our range of values depending on site and month (Fig. 3). This similarity supports the validity of these estimates and suggests that a DO-based approach is appropriate for calculating whole-ecosystem metabolism in the Delta.

Despite the ease of applicability of diel open water estimates, of concern in strongly tidal systems are longitudinal gradients in DO, which if present would invalidate the assumption of negligible advection in Eq. 1. Previous work has shown that longer term averaging helps to ameliorate this concern (Loken et al., 2021), which we applied in our study by averaging daily values to a monthly resolution. However, under certain conditions further corrections may be necessary, as evidenced by occasional positive estimates of R and negative estimates of GPP – ecologically impossible results that nevertheless are important to retain to avoid artificially inflating metabolic rates (Staehr et al., 2010). In our study, negative estimates of R accounted for ~ 10% of our results, particularly during the winter when advection is higher and metabolic rates are lower (Fig. 3). One way to account for advection is to fit a series of weighted regressions to remove its impact (Beck et al., 2015). This approach is complex to apply and may over-filter metabolic values, resulting in diminished estimates relative to true values (Beck et al., 2015). There were also instances in our dataset where neither long-term averaging nor tidal filtering are likely to ameliorate the impacts of advection. For example, during March–April 2017 and April-June 2019, estimates at FPT indicate positive R and negative GPP (Fig. 3A). FPT did not experience flow reversal during either period, so these results do not stem from asymmetrical tidal gradients in DO and as such would not be corrected using a tidal filtering approach. Rather, both periods were characterized by historically high precipitation in the Delta, which may have biased diel estimates of metabolism by altering patterns of discharge and residence times in the region. FPT is the furthest upstream station within the Sacramento River (Fig. 1), and it is possible that pulses of surface runoff to the Sacramento River resulted in substantial longitudinal gradients in DO that biased estimates even after monthly averaging. Nevertheless, future work to incorporate tidal filtering to address the impact of advection from Delta metabolic estimates could be beneficial. Another solution to account for DO advection is the deployment of a second DO sensor at a known distance from each mooring, allowing for the calculation of a true advective DO term (Gruber et al., 2017). However, given that resource managers often choose to maximize spatial coverage with limited instrumentation, any approach that involves multiple DO sensors at a single site may be infeasible for long-term monitoring efforts. Instead, it may be best to simply disregard estimates during abnormally high flow conditions in regions strongly influenced by surface runoff.

### Drivers of Delta Metabolism

Our results suggest mixed impacts of nutrients, and in particular DIN, on Delta metabolism. The largest point source of DIN to the Delta has historically been the EchoWater Facility, which from 2020–2021 underwent extensive renovations to decrease DIN outflow by > 3/4 while shifting the dominant species from NH_4_^+^ to NO_3_^−^ (Mussen et al., 2024). While decreasing nutrient outflows generally lowers ecosystem primary production, there was hope that switching effluent from predominantly NH_4_^+^ to NO_3_^−^ may enhance phytoplankton metabolism (Glibert, 2010), which is of important management concern as phytoplankton have historically formed the base of the Sacramento River food web (Cooke et al., 2018; Jassby, 2008). Overall, our results suggest minor impacts from EchoWater upgrades on metabolism, chl *a*, and possibly NO_3_^−^, with all three parameters demonstrating slight increases at WGA relative to FPT following upgrades (Fig. 5). These changes were spurred primarily by decreases at FPT – located upstream of EchoWater and thus presumably unrelated to plant upgrades– rather than increases at WGA which is located downstream. Metabolism at FPT also primarily decreased in the relatively low productivity spring months, rather than the late summer/early fall when values were highest (Fig. 5A). Likewise, although chl *a* levels at WGA were greater than at FPT following upgrades after being approximately equal before, these differences were small (~ 2 µg L^−1^), and were again driven by decreases at FPT with values at WGA showing no change (Fig. 5D). It is important to note that given the limited time since these upgrades went online it is difficult to tease apart the impacts of the upgrades against a dynamic physical environment. The three water years (WY19-WY21) immediately prior to the upgrades represent three of the four lowest flow years in our record, while the two years immediately following upgrades were wetter than normal (WY22-WY23) (Fig. 10). Nevertheless, our results suggest that ecosystem metabolism at WGA is either unchanged or mildly enhanced.

One confounding factor in quantifying the impact of EchoWater upgrades on ecosystem metabolism is the ability of DO-based measurements to separate out R from oxygen demand stemming from nitrification of EchoWater effluent. Prior to upgrades, nitrification along the reach of the Sacramento River between FPT and WGA was substantial, ranging from 1–4 mmol N m^−3^ d^−1^ depending on time of year (Kraus et al., 2017b). Assuming an average depth equal to the mean of the FPT and WGA cross-sections (7.75 m), these nitrification rates are equivalent to a oxygen demand of 15—60 mmol O_2_ m^−2^ d^−1^. Nitrification likely diminished substantially following upgrades, due to the loss of inputs of NH_4_^+^ from the WWTP. If nitrification significantly impacted our pre-upgrade metabolic estimates, this now lowered oxygen demand would manifest as apparent decreases to R at WGA, with GPP held constant. Instead, what changes that did occur to R at WGA were mirrored in GPP (Fig. 5B), suggesting that historic nitrification of NH_4_^+^ discharged by EchoWater likely occurred upstream of the measurement footprint of the WGA sensor, and that our estimates of DO uptake can rightfully to attributed to R. Nevertheless, it remains possible that changes to nitrification or biological oxygen demand spurred by the EchoWater upgrades may affect our interpretation of metabolic results.

Anthropogenic alterations to Delta hydrodynamics, including the deployment of EDBs, were shown to greatly affect ecosystem metabolism. One such EDB was placed near the JER mooring during a severe drought in 2021–2022, cutting off the sensor from the heavily vegetated Franks Tract and thus removing it from the measurement footprint. Impacts on JER metabolism were immediate, as both GPP and R both fell by ~ 50–60% during the period of EDB deployment (Fig. 8). Baseline seasonal NEM at JER is defined by positive values in the spring and early summer, followed by negative NEM in late summer and fall. This pattern is observable in 2019, 2020, and 2024 (Fig. 3E), and can be attributed to seasonal buildup and senescence of aquatic vegetation within Franks Tract. As with the Cache Slough complex described above, these metabolic patterns suggest that under baseline conditions OC in the form of macrophyte detritus produced within Franks Tract is advected out of the system, where it subsequently decomposes in deeper water areas of the Delta. During the period of the EDB deployment this pattern was broken, and NEM was consistently negative throughout. These changes to NEM, combined with decreased metabolism, indicates a loss in OC connectivity between the JER mooring and Franks Tract. Taken as a whole, our results again show that Delta metabolism is driven largely by benthic macrophytes, and that flow alterations can induce substantial changes to hydrologic OC transport in the region.

The largest anthropogenic alterations to Delta hydrodynamics are exports of freshwater to support agriculture in California’s Central Valley, which are managed using a complicated network of canals and pumps that include flow diversions from the Sacramento River through the DCC. The DCC is located near the WGA mooring, and when open, connects the Sacramento River with the Mokelumne River which ultimately flows into the central Delta, near Franks Tract (Fig. 1). Flow through the DCC is managed based on water availability and key species concerns (Lund, 2016), resulting in substantial interannual variations with likely impacts on ecosystem metabolism. Our results also suggest possible, albeit uncertain, positive effects of Q_DCC_ on the magnitudes of both R_WY_ and GPP_WY_ (Table 5), an effect largely driven by impacts on JER. Random effect slopes (Table S2) suggest that Q_DCC_ had nearly three times the impact on metabolism at JER compared with the other sites. Effects of Q_DCC_ were in the opposite direction of Q_FPT_ (Table 5), and may be attributed to increasing flow out of Franks Tract to the JER mooring. It is also possible diminished Q_DCC_ during the 2021–2022 drought contributed to the decreases in R and GPP observed at JER during EDB deployment, as this coincided with the lowest Q_DCC_ in our record (note: JER WY21 and WY22 results were omitted when computing LMM results). Interestingly, Q_DCC_ had no impact on metabolism at WGA (Table 5, Table S2), despite its proximity to the opening of the DCC. This is likely because Q_DCC_ scales with Q_FPT_, which was controlled for in our analysis.

T and Tu were both significant drivers of ecosystem metabolism after accounting for seasonal variation (Fig. 9, Table 2). Tu was only found to be a driver of GPP and not R, likely due to its impact on decreasing the photic depth and light availability for photosynthesis (Jassby & Platt, 1976). Both R and GPP were strongly correlated with T, as increased T is associated with enhanced microbial respiration (Apple et al., 2006), phytoplankton growth rates (Bissinger et al., 2008), and macrophyte respiration (Collier et al., 2017). A common parameter used to determine the impact of T on R is the Q_10_ value, defined as the proportional change in R predicted from an increase in T of 10 °C. Although this equation is typically fit using an exponential equation (Lloyd & Taylor, 1994), given the confounding impacts of turbidity and seasonality this formulation is not possible in our study. However, we can achieve a back-of-envelope estimate of Q_10_ from our LMM results by combining fixed effects (Table 2) and random effects (Table S1) results, which predict increases in the magnitude of R ranging from 70 (RYE) to 160 (JER) mmol m^−2^ d^−1^ per 10 °C in T. Assuming an increase in T from 10 to 20 °C – the approximate seasonal variation in the Delta –results in an equivalent Q_10_ of ~ 2.5–4 for FPT, WGA, LIB and JER, within the range of typical Q_10_ for estuaries (Lomas et al., 2002). Past studies of phytoplankton respiration in SFE have disregarded temperature as input for metabolism calculations (e.g. Jassby et al., 2002). The results of our study suggest that it may be important to revisit this assumption, and warrant further research of T impacts on water column metabolism in SFE.

As the earth continues to warm, California is likely to experience “wetter” wet years and “drier” dry years (Mallakpour et al., 2018), with major impacts on Delta metabolism. Our annual metabolic model – based on 10 + years of data – shows a negative correlation between Q_FPT_ (used as a proxy for WY precipitation) and the magnitudes of both R_WY_ and GPP_WY_ (Fig. 10). Diminished metabolism with increased precipitation has been shown in other estuaries (Tassone & Bukaveckas, 2019), and occurs despite a greater depth that should increase areal metabolism if per volume rates are held constant (Eq. 6). What is likely happening in the Delta and other systems is that increased water depth diminishes benthic GPP by limiting the amount of light that reaches the benthos (Koweek et al., 2018), while increased turbidity associated with surface runoff also limits both pelagic and benthic GPP by enhancing light limitation (Table 2). Our results are in contrast to a past study of Delta metabolism that suggested increased precipitation should enhance ecosystem metabolism, primarily by increasing the wetted area in the Yolo Bypass in the northern Delta (Cloern et al., 2021). In their study, Cloern et al. (2021) assumed a constant rate of areal production irrespective of precipitation, which our results suggest is not the case. Instead, the impact of drought on Delta metabolism is likely to involve a complex interplay between enhanced areal production and a smaller wetted area, with further research warranted to better elucidate whole-system impacts.

Taken as a whole, results from this study support our conceptual model of Delta metabolism (Fig. 2), showing how rates of ecosystem metabolism in the Delta depend upon the interplay between biota – including macrophytes, phytoplankton, and bacteria – with abiotic drivers such as nutrients, light, temperature, and hydrodynamics to produce and consume OC. All of these OC pools and metabolic drivers have been modified by, or are associated with, human activity in the Delta. As IAV dominates Delta C cycling, drivers that impact plant metabolism were particularly impactful on Delta metabolism, including light availability, temperature, and the seasonal growth and die-off of plant material. This build-up and senescence of IAV likely drives spatial patterns of Delta metabolism, with shallow, high-biomass areas such as the Cache Slough complex and Franks Tract producing OC that is remineralized elsewhere in deep-water channels. Changes to Delta hydrodynamics, including EDBs and exports to the Central Valley, disrupt this flow of OC, altering spatial patterns of Delta metabolism. Although phytoplankton are a minor contributor to metabolism under baseline conditions, during blooms their contribution to GPP substantially increases, producing labile OC that is later rapidly remineralized resulting in sharp increases in R and negative NEM. Our results inform Delta resource managers on ecosystem-scale impacts of disparate environmental stressors, adding to a growing body of literature demonstrating the value of metabolic calculations as an ecosystem monitoring tool (Jankowski et al., 2021). In doing so, this study also illustrates the importance of long-term continuous monitoring data, without which our inferences would not have been possible.

## Supplementary Information

Below is the link to the electronic supplementary material.

Supplementary file1 (DOCX 48 KB)

## Data Availability

All water quality data used in this study are publicly available at waterdata.usgs.gov. Atmospheric data are publicly available at https://cimis.water.ca.gov/.

## References

[CR1] Apple, J. K., Del Giorgio, P. A., & Kemp, W. M. (2006). Temperature regulation of bacterial production, respiration, and growth efficiency in a temperate salt-marsh estuary. *Aquatic Microbial Ecology,**43*(3), 243–254. 10.3354/ame043243

[CR2] Bailey, K., Steinberg, C., Davies, C., Galibert, G., Hidas, M., McManus, M. A., Murphy, T., Newton, J., Roughan, M., & Schaeffer, A. (2019). Coastal mooring observing networks and their data products: Recommendations for the next decade. *Frontiers in Marine Science,**6*(APR), 1–22. 10.3389/fmars.2019.00180

[CR3] Beck, M. W., Hagy, J. D., & Murrell, M. C. (2015). Improving estimates of ecosystem metabolism by reducing effects of tidal advection on dissolved oxygen time series. *Limnology and Oceanography: Methods,**13*(12), 731–745. 10.1002/lom3.10062

[CR4] Berger, A. C., Berg, P., McGlathery, K. J., & Delgard, M. L. (2020). Long-term trends and resilience of seagrass metabolism: A decadal aquatic eddy covariance study. *Limnology and Oceanography,**65*(7), 1423–1438. 10.1002/lno.11397

[CR5] Bissinger, J. E., Montagnes, D. J. S., Sharples, J., & Atkinson, D. (2008). Predicting marine phytoplankton maximum growth rates from temperature: Improving on the Eppley curve using quantile regression. *Limnology and Oceanography,**53*(2), 487–493. 10.4319/lo.2008.53.2.0487

[CR6] Bouma-Gregson, K., Bosworth, D., Flynn, T., Maguire, A., Rinde, J., & Hartman, R. (2024). Delta Blue(green)s: The Impact of Drought and Drought Management Actions on Microcystis in the Sacramento–San Joaquin Delta. *San Francisco Estuary and Watershed Science*, *22*(1). 10.15447/sfews.2024v22iss1art2

[CR7] Boyer, K., & Sutula, M. (2015). *Factors Controlling Submersed and Floating Macrophytes in the Sacramento-San Joaquin Delta*. Southern California Coastal Water Research Project. Technical Report No. 870. Costa Mesa, CA.

[CR8] Boyer, K., Khanna, S., & Patten, M. (2023). Landscape transformation and variation in invasive species abundance drive change in primary production of aquatic vegetation in the sacramento–San Joaquin Delta. *San Francisco Estuary and Watershed Science*, *20*(4). 10.15447/sfews.2023v20iss4art2

[CR9] Caffrey, J. M. (2003). Production, respiration and net ecosystem metabolism U.S. estuaries. *Environmental Monitoring and Assessment,**81*(1–3), 207–219. 10.1023/A:1021385226315 12620017

[CR10] Caffrey, J. M. (2004). Factors controlling net ecosystem metabolism in U. S. estuaries. *Estuaries,**27*(1), 90–101.

[CR11] California Irrigation Management Information System (2025). Accessed Mar 20 2025 at https://cimis.water.ca.gov/

[CR12] Christman, M. A., Khanna, S., Drexler, J. Z., & Young, M. J. (2023). Ecology and ecosystem effects of submerged and floating aquatic vegetation in the sacramento-San Joaquin Delta. *San Francisco *Estuary* and Watershed Science, 20(4)*. 10.15447/sfews.2023v20iss4art3

[CR13] Cloern, J. E. (2001). Our evolving conceptual model of the coastal eutrophication problem. *Marine Ecology Progress Series,**210*, 223–253.

[CR14] Cloern, J. E., Foster, S. Q., & Kleckner, A. E. (2014). Phytoplankton primary production in the world’s estuarine-coastal ecosystems. *Biogeosciences,**11*(9), 2477–2501. 10.5194/bg-11-2477-2014

[CR15] Cloern, J. E., Jassby, A. D., Schraga, T. S., Nejad, E., & Martin, C. (2017). Ecosystem variability along the estuarine salinity gradient: Examples from long-term study of San Francisco Bay. *Limnology and Oceanography,**62*, S272–S291. 10.1002/lno.10537

[CR16] Cloern, J. E., Safran, S. M., Smith Vaughn, L., Robinson, A., Whipple, A. A., Boyer, K. E., Drexler, J. Z., Naiman, R. J., Pinckney, J. L., Howe, E. R., Canuel, E. A., & Grenier, J. L. (2021). On the human appropriation of wetland primary production. *Science of the Total Environment,**785*, 147097. 10.1016/j.scitotenv.2021.147097

[CR17] Cloern, J. E., Schraga, T. S., Nejad, E., & Martin, C. (2020). Nutrient status of san francisco bay and its management implications. *Estuaries and Coasts,**43*(6), 1299–1317. 10.1007/s12237-020-00737-w

[CR18] Cohen, R. A., Wilkerson, F. P., Parker, A. E., & Carpenter, E. J. (2014). Ecosystem-scale rates of primary production within wetland habitats of the northern San Francisco Estuary. *Wetlands,**34*(4), 759–774. 10.1007/s13157-014-0540-3

[CR19] Cole, J. J., & Caraco, N. F. (1998). Atmospheric exchange of carbon dioxide in a low-wind oligotrophic lake measured by the addition of SF6. *Limnology and Oceanography,**43*(4), 647–656. 10.4319/lo.1998.43.4.0647

[CR20] Cole, J. J., Pace, M. L., Carpenter, S. R., & Kitchell, J. F. (2000). Persistence of net heterotrophy in lakes during nutrient addition and food web manipulations. *Limnology and Oceanography,**45*(8), 1718–1730. 10.4319/lo.2000.45.8.1718

[CR21] Collier, C. J., Ow, Y. X., Langlois, L., Uthicke, S., Johansson, C. L., O’Brien, K. R., Hrebien, V., & Adams, M. P. (2017). Optimum temperatures for net primary productivity of three tropical seagrass species. *Frontiers in Plant Science,**8*(August), 1–14. 10.3389/fpls.2017.01446 PMC5572403 28878790

[CR22] Cooke, R. G., Joab, C., & Lu, Z. (2018). *Delta Nutrient Research Plan*. Regional Water Quality Control Board, Central Valley Region, California Environmental Protection Agency.

[CR23] Durand, J., Bombardelli, F., Fleenor, W., Henneberry, Y., Herman, J., Jeffres, C., Leinfelder-Miles, M., Lusardi, R., Manfree, A., Medellín-Azura, J., Milligan, B., Moyle, P., & Lund, J. (2020). Drought and the Sacramento–San Joaquin Delta, 2012–2016: Environmental Review and Lessons. *San Francisco Estuary and Watershed Science*, *18*(2). 10.15447/sfews.2020v18iss2art2

[CR24] Elser, J. J., Bracken, M. E. S., Cleland, E. E., Gruner, D. S., Harpole, W. S., Hillebrand, H., Ngai, J. T., Seabloom, E. W., Shurin, J. B., & Smith, J. E. (2007). Global analysis of nitrogen and phosphorus limitation of primary producers in freshwater, marine and terrestrial ecosystems. *Ecology Letters,**10*(12), 1135–1142. 10.1111/j.1461-0248.2007.01113.x 17922835

[CR25] Gattuso, J. P., Frankignoulle, M., & Wollast, R. (1998). Carbon and carbonate metabolism in coastal aquatic ecosystems. *Annual Review of Ecology and Systematics,**29*, 405–434. 10.1146/annurev.ecolsys.29.1.405

[CR26] Glibert, P. M. (2010). Long-term changes in nutrient loading and stoichiometry and their relationships with changes in the food web and dominant pelagic fish species in the San Francisco Estuary. *California. Reviews in Fisheries Science,**18*(2), 211–232. 10.1080/10641262.2010.492059

[CR28] Glud, R. N. (2008). Oxygen dynamics of marine sediments. *Marine Biology Research,**4*(4), 243–289. 10.1080/17451000801888726

[CR29] Gross, E., Holleman, R., Kimmerer, W., Kraus, T., Bergamaschi, B., Burdick-Yahya, S., & Senn, D. (2024). Estimating biogeochemical rates using a computationally efficient Lagrangian approach. *Estuaries and Coasts,**47*(6), 1435–1455. 10.1007/s12237-024-01381-4

[CR30] Gruber, R. K., Lowe, R. J., & Falter, J. L. (2017). Metabolism of a tide-dominated reef platform subject to extreme diel temperature and oxygen variations. *Limnology and Oceanography,**62*(4), 1701–1717. 10.1002/lno.10527

[CR31] Hansen, J. C. R., & Reidenbach, M. A. (2012). Wave and tidally driven flows in eelgrass beds and their effect on sediment suspension. *Marine Ecology Progress Series,**448*, 271–287. 10.3354/meps09225

[CR32] Hartman, R., Rasmussen, N., Bosworth, D., Berg, M., Ateljevich, E., Flynn, T., Wolf, B., Pennington, T., & Khanna, S. (2022). Temporary Urgency Change Petition of 2021 and Emergency Drought Salinity Barrier: Impact on Harmful Algal Blooms and Aquatic Weeds in the Delta. Sacramento (CA): California Department of Water Resources. May 2022. 188 pp. + appendix.

[CR33] Hestir, E. L., Schoellhamer, D. H., Greenberg, J., Morgan-King, T., & Ustin, S. L. (2016). The effect of submerged aquatic vegetation expansion on a declining turbidity trend in the Sacramento-San Joaquin River Delta. *Estuaries and Coasts,**39*(4), 1100–1112. 10.1007/s12237-015-0055-z

[CR34] Jähne, B. J., Münnich, K. O. M., Bösinger, R., Dutzi, A., Huber, W., & Libner, P. (1987). On the parameters influencing air-water gas exchange. *Journal of Geophysical Research,**92*(C2), 1937–1949.

[CR35] Jankowski, K. J., Mejia, F. H., Blaszczak, J. R., & Holtgrieve, G. W. (2021). Aquatic ecosystem metabolism as a tool in environmental management. *Wiley Interdisciplinary Reviews: Water,**8*(4), Article e1521. 10.1002/wat2.1521

[CR36] Jassby, A. D. (2008). Phytoplankton in the Upper San Francisco Estuary: Recent Biomass Trends, Their Causes, and Their Trophic Significance. *San Francisco Estuary and Watershed Science*, *6*(1). 10.15447/sfews.2008v6iss1art2

[CR37] Jassby, A. D., & Cloern, J. E. (2000). Organic matter sources and rehabilitation of the Sacramento-San Joaquin Delta (California, USA). *Aquatic Conservation: Marine and Freshwater Ecosystems,**10*(5), 323–352. 10.1002/1099-0755(200009/10)10:5>253C323::AID-AQC417>253E3.0.CO;2-J

[CR38] Jassby, A. D., Cloern, J. E., & Cole, B. E. (2002). Annual primary production: Patterns and mechanisms of change in a nutrient-rich tidal ecosystem. *Limnology and Oceanography,**47*(3), 698–712. 10.4319/lo.2002.47.3.0698

[CR39] Jassby, A. D., & Platt, T. (1976). Mathematical formulation of the relationship between photosynthesis and light for phytoplankton. *Limnology and Oceanography,**21*(4), 540–547. 10.4319/lo.1976.21.4.0540

[CR40] Jungbluth, M., Lee, C., Patel, C., Ignoffo, T., Bergamaschi, B., & Kimmerer, W. (2021). Production of the copepod pseudodiaptomus forbesi is not enhanced by ingestion of the diatom aulacoseira granulata during a bloom. *Estuaries and Coasts,**44*(4), 1083–1099. 10.1007/s12237-020-00843-9

[CR41] Khanna, S., Santos, M. J., Boyer, J. D., Shapiro, K. D., Bellvert, J., & Ustin, S. L. (2018). Water primrose invasion changes successional pathways in an estuarine ecosystem. *Ecosphere*. 10.1002/ecs2.2418

[CR42] Kimmerer, W. J., & Thompson, J. K. (2014). Phytoplankton growth balanced by clam and zooplankton grazing and net transport into the low-salinity zone of the San Francisco Estuary. *Estuaries and Coasts,**37*(5), 1202–1218. 10.1007/s12237-013-9753-6

[CR43] Koopmans, D. J., & Berg, P. (2015). Stream oxygen flux and metabolism determined with the open water and aquatic eddy covariance techniques. *Limnology and Oceanography,**60*, 1344–1355. 10.1002/lno.10103

[CR44] Koweek, D. A., Zimmerman, R. C., Hewett, K. M., Gaylord, B., Giddings, S. N., Nickols, K. J., Ruesink, J. L., Stachowicz, J. J., Takeshita, Y., & Caldeira, K. (2018). Expected limits on the ocean acidification buffering potential of a temperate seagrass meadow. *Ecological Applications,**28*(7), 1694–1714. 10.1002/eap.1771 30063809

[CR45] Kraus, T. E. C., Bergamaschi, B. A., & Downing, B. D. (2017a). An introduction to high-frequency nutrient and biogeochemical monitoring for the Sacramento–San Joaquin Delta, northern California: U.S. Geological Survey Scientific Investigations Report 2017–5071, 41 p., 10.3133/sir20175071

[CR46] Kraus, T. E. C., O’Donnell, K., Downing, B. D., Burau, J. R., & Bergamaschi, B. A. (2017b). Using paired in situ high frequency nitrate measurements to better understand controls on nitrate concentrations and estimate nitrification rates in a wastewater-impacted river. *Water Resources Research,**53*(10), 8423–8442. 10.1002/2017WR020670

[CR47] Krause-Jensen, D., Markager, S., & Dalsgaard, T. (2012). Benthic and pelagic primary production in different nutrient regimes. *Estuaries and Coasts,**35*(2), 527–545. 10.1007/s12237-011-9443-1

[CR48] Lacy, J. R., Foster‐Martinez, M. R., Allen, R. M., & Drexler, J. Z. (2021). Influence of invasive submerged aquatic vegetation (*E. densa*) on currents and sediment transport in a freshwater tidal system. *Water Resources Research,**57*(8), Article e2020WR028789. 10.1029/2020WR028789

[CR49] Lloyd, J., & Taylor, J. A. (1994). On the temperature dependence of soil respiration. *Functional Ecology,**8*(3), 315–323.

[CR50] Loken, L. C., Van Nieuwenhuyse, E. E., Dahlgren, R. A., Lenoch, L. E. K., Stumpner, P. R., Burau, J. R., & Sadro, S. (2021). Assessment of multiple ecosystem metabolism methods in an estuary. *Limnology and Oceanography: Methods,**19*(11), 741–757. 10.1002/lom3.10458

[CR51] Lomas, M. W., Glibert, P. M., Shiah, F. K., & Smith, E. M. (2002). Microbial processes and temperature in Chesapeake Bay: Current relationships and potential impacts of regional warming. *Global Change Biology,**8*(1), 51–70. 10.1046/j.1365-2486.2002.00454.x

[CR52] Lopez, C. B., Cloern, J. E., Schraga, T. S., Little, A. J., Lucas, L. V., Thompson, J. K., & Burau, J. R. (2006). Ecological Values of Shallow-Water Habitats: Implications for the Restoration of Disturbed Ecosystems. *Ecosystems,**9*(3), 422–440. 10.1007/s10021-005-0113-7

[CR53] Lund, J. (2016). California’s Agricultural and Urban Water Supply Reliability and the Sacramento–San Joaquin Delta. *San Francisco Estuary and Watershed Science*, *14*(3). 10.15447/sfews.2016v14iss3art6

[CR54] Mallakpour, I., Sadegh, M., & AghaKouchak, A. (2018). A new normal for streamflow in California in a warming climate: Wetter wet seasons and drier dry seasons. *Journal of Hydrology,**567*, 203–211. 10.1016/j.jhydrol.2018.10.023

[CR55] Mann, M. E., & Gleick, P. H. (2015). Climate change and California drought in the 21st century. *Proceedings of the National Academy of Sciences of the United States of America,**112*(13), 3858–3859. 10.1073/pnas.1503667112 PMC4386383 25829537

[CR56] Moyle, P. B., Brown, L. R., Durand, J. R., & Hobbs, J. A. (2016). Delta smelt: Life history and decline of a once-abundant species in the San Francisco estuary. *San Francisco Estuary and Watershed Science*, *14*(2). 10.15447/sfews.2016v14iss2art6

[CR77] Mussen, T.D., Mine Berg, G., Driscall, S., Nordin, J.D., & Thompson, L. C. (2024) Clams on stilts: a phytoplankton bioassay investigating effects of wastewater effluent amendments and *Corbicula fluminea* grazing. *Aquatic Biology* 33, 13–31. 10.3354/ab00764

[CR57] Nardelli, S. C., Bouma-Gregson, K., Senn, D. B., Killam, D., Chelsky, A., Nejad, E., Richardson, E. T., Otten, T. G., Kraus, T. E. C., & Bergamaschi, B. A. (2026). Assessing environmental drivers and protist community dynamics that shaped the historic August 2022 heterosigma akashiwo bloom in San Francisco Bay, California. *Estuaries and Coasts,**49*(2), 39. 10.1007/s12237-025-01629-7

[CR58] Newey, W. K., & West, K. D. (1987). A simple, positive semi-definite, heteroskedasticity and autocorrelation consistent covariance matrix. *Econometrica,**55*(3), 703–708.

[CR59] Odum, H. T. (1956). Primary production in flowing waters. *Limnology and Oceanography,**1*(2), 102–117. 10.4319/lo.1956.1.2.0102

[CR60] Paerl, H. W. (2009). Controlling eutrophication along the freshwater-marine continuum: Dual nutrient (N and P) reductions are essential. *Estuaries and Coasts,**32*(4), 593–601. 10.1007/s12237-009-9158-8

[CR61] Pinckney, J. L., Paerl, H. W., Tester, P., & Richardson, T. L. (2001). The role of nutrient loading and eutrophication in estuarine ecology. *Environmental Health Perspectives,**109*(SUPPL. 5), 699–706. 10.1289/ehp.01109s5699 PMC1240600 11677178

[CR62] Reichert, P., Uehlinger, U., & Acuña, V. (2009). Estimating stream metabolism from oxygen concentrations: Effect of spatial heterogeneity. *Journal of Geophysical Research: Biogeosciences,**114*, Article G03016. 10.1029/2008JG000917

[CR63] Robinson, A., Richey, A., Cloern, J. E., Boyer, K. E., Burau, J. R., Canuel, E. A., Drexler, J. Z., DeGeorge, J. F., Howe, E. R., Kneib, R. T., Mueller-Solger, A., Pinckney, J. L., Naiman, R. J., Schoellhamer, D. H., Simenstad, C. A., Drexler, J. Z., Grenier, L., Grossinger, R., Howe, E. R., … Simenstad, C. A. (2016). Primary production in the Sacramento-San Joaquin Delta: A science strategy to quantify change and identify future potential. *A Report from SFEI-ASC’s Resilient Landscapes Program*, (781). http://www.sfei.org/projects/delta-landscapes-project

[CR64] Senn, D. B., Kraus, T., Richey, A., Bergamaschi, B. A., Brown, L., Conrad, L., Francis, C., Kimmerer, W. J., Kudela, R., Otten, T. G., Parker, A. E., Robinson, A., Mueller-Solger, A., Stern, D., & Thompson, J. (2020). Changing Nitrogen Inputs to the northern San Francisco Estuary: Science Opportunities and Constraints. SFEI Contribution No. 973. San Francisco Estuary Institute: Richmond, CA.

[CR65] Smits, A. P., Scordo, F., Tang, M., Cortés, A., Farruggia, M. J., Culpepper, J., Chandra, S., Jin, Y., Valbuena, S. A., Watanabe, S., Schladow, G., & Sadro, S. (2024). Wildfire smoke reduces lake ecosystem metabolic rates unequally across a trophic gradient. *Communications Earth & Environment,**5*(1), Article 265. 10.1038/s43247-024-01404-9

[CR66] Sobczak, W. V., Cloern, J. E., Jassby, A. D., Cole, B. E., Schraga, T. S., & Arnsberg, A. (2005). Detritus fuels ecosystem metabolism but not metazoan food webs in San Francisco estuary’s freshwater delta. *Estuaries,**28*(1), 124–137. 10.1007/BF02732759

[CR67] Staehr, P. A., Bade, D., Koch, G. R., Williamson, C., Hanson, P. C., Cole, J. J., & Kratz, T. (2010). Lake metabolism and the diel oxygen technique: State of the Science. *Limnology and Oceanography: Methods,**8*, 628–644. 10.4319/lom.2010.8.628

[CR68] Staehr, P. A., Testa, J. M., Kemp, W. M., Cole, J. J., Sand-Jensen, K., & Smith, S. V. (2012). The metabolism of aquatic ecosystems: History, applications, and future challenges. *Aquatic Sciences,**74*(1), 15–29. 10.1007/s00027-011-0199-2

[CR69] Statham, P. J. (2012). Nutrients in estuaries – An overview and the potential impacts of climate change. *Science of the Total Environment,**434*, 213–227. 10.1016/j.scitotenv.2011.09.088 22119025

[CR70] Ta, J., Anderson, L. W. J., Christman, M. A., Khanna, S., Kratville, D., Madsen, J. D., Moran, P. J., & Viers, J. H. (2017). Invasive aquatic vegetation management in the Sacramento-San Joaquin River Delta: Status and recommendations. *San Francisco Estuary and Watershed Science*, *15*(4). 10.15447/sfews.2017v15iss4art5

[CR71] Tassone, S. J., & Bukaveckas, P. A. (2019). Seasonal, interannual, and longitudinal patterns in estuarine metabolism derived from diel oxygen data using multiple computational approaches. *Estuaries and Coasts,**42*(4), 1032–1051. 10.1007/s12237-019-00526-0

[CR72] U.S. Geological Survey (2025) USGS Water Data for the Nation: U.S. Geological Survey National Water Information System database. Accessed April 24 2025 at 10.5066/F7P55KJN

[CR73] Vaughn, L. S., Safran, S., Robinson, A., Whipple, A., Richey, A., Grenier, L., Cloern, J., Andrews, S., Boyer, K., Drexler, J., Howe, E., Naiman, R., Patten, M., & Pinckney, J. (2020). Delta Landscapes Primary Production: Past, Present, Future. Prepared for the Delta Stewardship Council. A Report of SFEI-ASC’s Resilient Landscapes Program, Publication #988, San Francisco Estuary Institute-Aquatic Science Center, Richmond, CA.

[CR74] Wanninkhof, R. (1992). Relationship between wind speed and gas exchange over the ocean. *Journal of Geophysical Research,**97*(C5), 7373–7382. 10.1029/92JC00188

[CR75] Williams, S. L., & Grosholz, E. D. (2008). The invasive species challenge in estuarine and coastal environments: Marrying management and science. *Estuaries and Coasts,**31*(1), 3–20. 10.1007/s12237-007-9031-6

[CR76] Young, R. G., Matthaei, C. D., & Townsend, C. R. (2008). Organic matter breakdown and ecosystem metabolism: Functional indicators for assessing river ecosystem health. *Journal of the North American Benthological Society,**27*(3), 605–625. 10.1899/07-121.1

