# Supplementary material for: Drivers of Ecosystem Metabolism in an Anthropogenically Modified Delta
Source: Estuaries Coast. 2026 Oct 23;50(1):13. doi: 10.1007/s12237-026-01833-z (PMC13694349; doi:10.1007/s12237-026-01833-z)
Supplement: Supplementary file 1 [file 12237_2026_1833_MOESM1_ESM.docx]

Table S1: Summary of site-specific random effects for monthly respiration (R) and gross primary production (GPP) linear mixed effect models, based on temperature (T) and turbidity (Tu).

Table S2: Summary of site-specific random effects for water year respiration (R_WY_) and gross primary production (GPP_WY_) linear mixed effect models, based on discharge at Freeport and through the Delta Cross Channel (Q_FPT_ and Q_DCC_, respectively).
